# *Salmonella enterica* Serovar Typhi Conceals the Invasion-Associated Type Three Secretion System from the Innate Immune System by Gene Regulation

**DOI:** 10.1371/journal.ppat.1004207

**Published:** 2014-07-03

**Authors:** Sebastian E. Winter, Maria G. Winter, Victor Poon, A. Marijke Keestra, Torsten Sterzenbach, Franziska Faber, Luciana F. Costa, Fabiane Cassou, Erica A. Costa, Geraldo E. S. Alves, Tatiane A. Paixão, Renato L. Santos, Andreas J. Bäumler

**Affiliations:** 1 Department of Medical Microbiology and Immunology, School of Medicine, University of California, Davis, Davis, California, United States of America; 2 Departamento de Patologia Geral, Instituto de Ciências Biológicas, Universidade Federal de Minas Gerais, Belo Horizonte, Minas Gerais, Brazil; 3 Departamento de Clinica e Cirurgia Veterinárias, Escola de Veterinária, Universidade Federal de Minas Gerais, Belo Horizonte, Minas Gerais, Brazil; Stanford University School of Medicine, United States of America

## Abstract

Delivery of microbial products into the mammalian cell cytosol by bacterial secretion systems is a strong stimulus for triggering pro-inflammatory host responses. Here we show that *Salmonella enterica* serovar Typhi (*S.* Typhi), the causative agent of typhoid fever, tightly regulates expression of the invasion-associated type III secretion system (T3SS-1) and thus fails to activate these innate immune signaling pathways. The *S.* Typhi regulatory protein TviA rapidly repressed T3SS-1 expression, thereby preventing RAC1-dependent, RIP2-dependent activation of NF-κB in epithelial cells. Heterologous expression of TviA in *S. enterica* serovar Typhimurium (*S.* Typhimurium) suppressed T3SS-1-dependent inflammatory responses generated early after infection in animal models of gastroenteritis. These results suggest that *S.* Typhi reduces intestinal inflammation by limiting the induction of pathogen-induced processes through regulation of virulence gene expression.

## Author Summary

Bacterial pathogens translocate effector proteins into the cytoplasm of host cells to manipulate the mammalian host. These processes, e.g. the stimulation of small regulatory GTPases, activate the innate immune system and induce pro-inflammatory responses aimed at clearing invading microbes from the infected tissue. Here we show that strict regulation of virulence gene expression can be used as a strategy to limit the induction of inflammatory responses while retaining the ability to manipulate the host. Upon entry into host tissue, *Salmonella enterica* serovar Typhi, the causative agent of typhoid fever, rapidly represses expression of a virulence factor required for entering tissue to avoid detection by the host innate immune surveillance. This tight control of virulence gene expression enables the pathogen to deploy a virulence factor for epithelial invasion, while preventing the subsequent generation of pro-inflammatory responses in host cells. We conclude that regulation of virulence gene expression contributes to innate immune evasion during typhoid fever by concealing a pattern of pathogenesis.

## Introduction

One function of the innate immune system in the intestinal tract is to generate temporary inflammatory responses against invasive enteric pathogens while avoiding detrimental overreaction against harmless commensal bacteria under homeostatic conditions. In contrast to commensal microbes, pathogenic microbes express an array of virulence factors to manipulate host cell functions. Pathogen-induced processes, also known as patterns of pathogenesis [1], activate specific pathways of the innate immune system, enabling the host to distinguish virulent microbes from ones with lower disease-causing potential. By detecting pathogen-induced processes the host can escalate innate immune responses to levels that are appropriate to the threat [2].

*Salmonella enterica* serovar Typhimurium (*S.* Typhimurium), an invasive enteric pathogen associated with human gastroenteritis, triggers acute intestinal inflammation in the terminal ileum and colon, thereby producing symptoms of diarrhea and abdominal pain within less than one day after ingestion [3]. The inflammatory infiltrate in the affected intestinal tissue is dominated by neutrophils [4], [5]. Similarly, neutrophils are the primary cell type in the stool during acute illness [6]–[8]. In contrast, individuals infected with serovar Typhi (*S.* Typhi) develop a febrile illness (typhoid fever) with systemic dissemination of the organism. In contrast to *Salmonella*-induced gastroenteritis, only a third of patients develop diarrhea that is characterized by a dominance of mononuclear cells in the stool [6]. The dominant cell type in intestinal infiltrates is mononuclear, while neutrophils are infrequent [9]–[11]. Unlike *S.* Typhi, interaction of *S.* Typhimurium with intestinal model epithelia induces hepoxilin A3-dependent transmigration of neutrophils [12]. Moreover, infection of human colonic tissue explants with *S.* Typhimurium results in the increased production of the neutrophil-attracting chemokine IL-8, while *S.* Typhi does not elicit this response [13]. These observations suggest that invasion of the intestinal mucosa by *S.* Typhimurium is accompanied by a rapid escalation of host responses leading to acute, purulent inflammation, while *S.* Typhi elicits little intestinal inflammation during early stages of infection, however the molecular mechanisms underlying these apparent differences are poorly defined.

One pathogen-induced processes that triggers pro-inflammatory immune responses is the transfer of bacterial molecules into the host cell cytosol by secretion systems. The invasion-associated type III secretion system (T3SS-1) expressed by all *Salmonella* serovars and delivers effector proteins into the cytosol of epithelial cells [14]. A subset of these translocated effector proteins activate Rho-family GTPases [15]–[18], thereby triggering alterations in the host cell cytoskeleton that result in bacterial invasion of epithelial cells [19]. Excessive stimulation of Rho-family GTPases activates the transcription factor nuclear factor kappa-light-chain-enhancer of activated B cells (NF-κB) and promotes the subsequent release of proinflammatory cytokines and chemokines [15], [20], [21]. In a bovine model of *S.* Typhimurium-induced gastroenteritis, the rapid induction of intestinal inflammation and diarrhea requires the T3SS-1 apparatus as well as the effector proteins SipA, SopA, SopB, SopD, and SopE2 [22]–[24]. Similarly, in a murine model of *Salmonella* induced colitis, SipA, SopE and SopE2 can independently induce intestinal inflammation [25] and mutants lacking a functional T3SS-1 are unable to initiate neutrophil recruitment to the intestinal mucosa during early infection [25], [26]. These findings indicate that T3SS-1-mediated effector translocation induces innate immune responses during *S.* Typhimurium-induced colitis.

Similar to *S.* Typhimurium, invasion of cultured intestinal epithelial cells by *S.* Typhi is mediated by the T3SS-1 [27]. Replacement of *S.* Typhimurium T3SS-1 effector proteins with their *S.* Typhi orthologues does not attenuate inflammatory responses elicited by *S.* Typhimurium in the intestinal mucosa of calves [28], demonstrating that *S.* Typhi T3SS-1 effector proteins can exhibit intrinsic pro-inflammatory properties *in vivo*. Thus, the molecular basis for the absence of T3SS-1-dependent innate immune responses early during *S.* Typhi infection remains unclear.

## Results

### In contrast to *S.* Typhimurium, *S.* Typhi fails to activate the NF-κB signaling pathway in human epithelial cells

**Figure 1:**
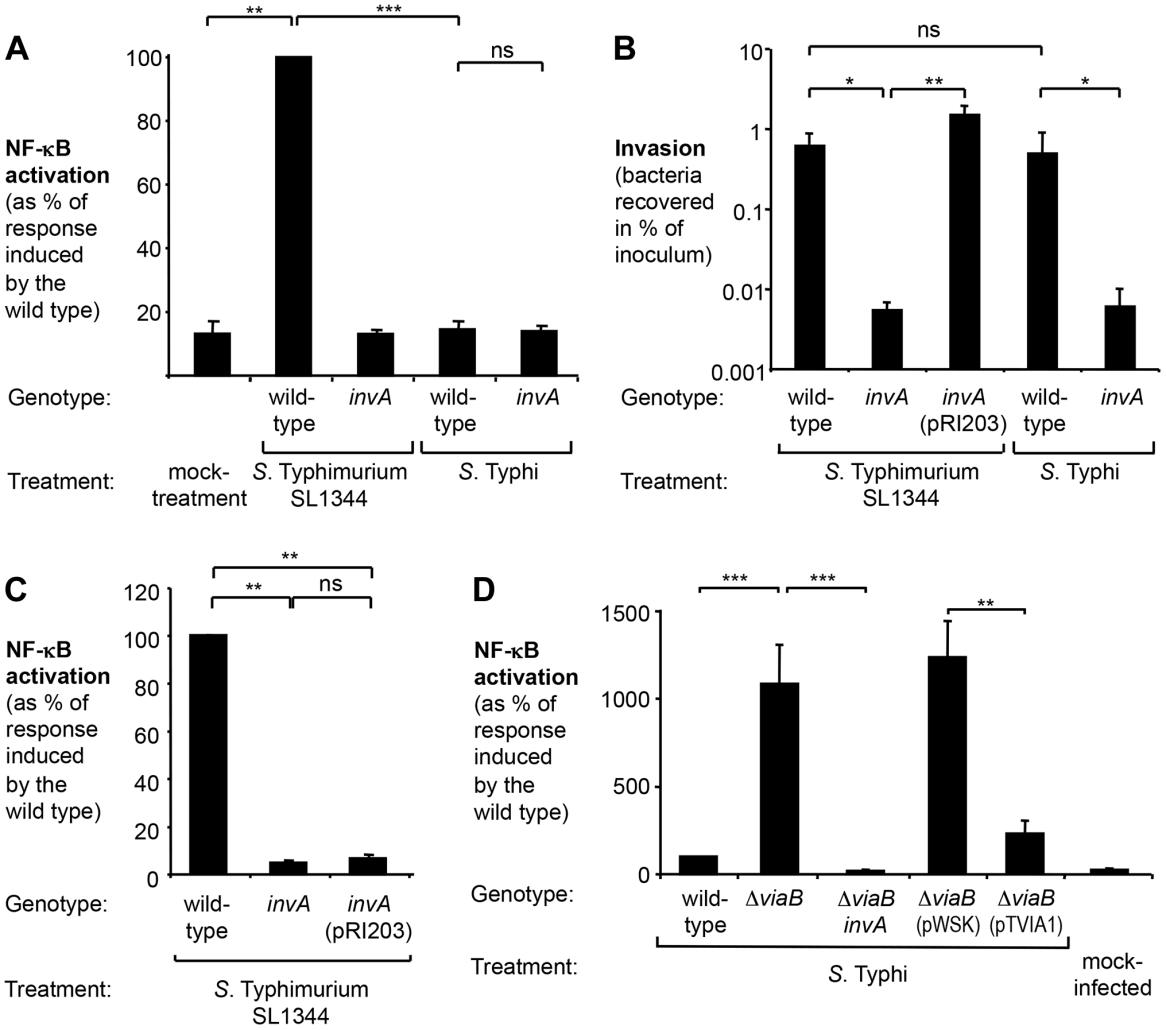
*S.* Typhi does not elicit inflammatory responses in epithelial cells. Human epithelial cells permanently transfected with a NF-κB-luciferase reporter system (HeLa 57A) were infected with the indicated *Salmonella* strains at a multiplicity of infection of 5 or mock treated (bacterial growth media alone). (A) Cells were infected with the *S.* Typhimurium wild type SL1344, an isogenic *invA* mutant (SW767), the *S.* Typhi wild type Ty2, an isogenic *invA* mutant (SW222). Luciferase activity as a measure of NF-κB activation was determined after 5 h (N = 3). (B) Monolayers of cells were infected for 1 h with the indicated *Salmonella* strains. Bacterial numbers recovered after 90 min of Gentamicin treatment were standardized to the number of the bacteria in the inoculum (N = 4). Plasmid pRI203 encodes the *Y. pseudotuberculosis* invasin. (C) Luciferase activity exhibited by *Salmonella*-infected HeLa57A cells was determined as described above (N = 4). (D) Cells were infected the *S.* Typhi wild-type strain Ty2, a Δ*viaB* mutant (SW347), an *invA* Δ*viaB* mutant (STY4), a Δ*viaB* mutant (STY2) harboring the cloning plasmid pWSK29 (pWSK), a Δ*viaB* mutant (STY2) expressing the *tviA* gene (pTVIA1) and luciferase activity determined 5 h after infection (N = 5). Bars represent geometric means ± standard error. *, *P*<0.05; **, *P*<0.01; ***, *P*<0.001; ns, not statistically significant.

To study the induction of pro-inflammatory signaling pathways upon infection with *S.* Typhimurium and *S.* Typhi, we employed a human epithelial cell line permanently transfected with a NF-κB-dependent luciferase reporter (HeLa 57A) [29]. Infection with the *S.* Typhimurium wild-type strain SL1344 resulted in a significant increase (7-fold; *P*<0.01) in luciferase activity compared to mock-infected cells (Fig. 1A), while a derivative of *S.* Typhimurium SL1344 carrying a mutation in the T3SS-1 apparatus gene *invA* (SW767) did not elicit NF-κB signaling [20], [30]. In contrast to the *S.* Typhimurium wild type, the *S.* Typhi wild-type strain Ty2 failed to trigger NF-κB activation (Fig. 1A), suggesting that *S.* Typhi is a poor activator of T3SS-1-dependent inflammatory processes in human epithelial cells.

### Effect of the *viaB* operon on T3SS-1 mediated inflammatory responses in epithelial cells

The T3SS-1 mediates invasion of non-phagocytic cells. *S.* Typhi has been reported to differ from *S.* Typhimurium with regards to invasion of human epithelial cells [31]–[33], thus raising the possibility that the observed differential activation of the NF-κB signaling pathway could be due to varying degrees of invasiveness. To test this hypothesis, HeLa cells were infected with *S.* Typhimurium and *S.* Typhi strains and a gentamicin protection assay was performed (Fig. 1B). The *S.* Typhimurium wild type SL1344 and the *S.* Typhi wild type Ty2 were recovered in similar numbers, while the respective isogenic *invA*-deficient mutants displayed significantly reduced invasiveness. T3SS-1 activity has to two functional consequences: manipulation of host signaling pathways and subsequent bacterial uptake. To discern between effects mediated directly by the T3SS-1 or indirectly by increasing the intracellular bacterial load, we next sought to reinstate invasiveness of the *S.* Typhimurium *invA* mutant without restoring T3SS-1 function. Expression of the *Yersinia pseudotuberculosis* invasin, encoded by the plasmid pRI203, raised invasiveness of the *S.* Typhimurium *invA* mutant comparable to the wild type strain (Fig. 1B), but failed to restore the ability to induce NF-κB activation in epithelial cells (Fig. 1C) [34]. Taken together, these observations indicate that immune evasion by *S.* Typhi did not directly correlate with the intracellular bacterial load or invasiveness.

Despite causing disparate disease entities, the genomes of *S.* Typhimurium and *S.* Typhi display remarkable similarity. Chromosomal DNA sequences of both serovars are highly syntenic, with mostly minor inversions, deletions and insertions [35], [36]. One DNA region that is present in *S.* Typhi but absent from *S.* Typhimurium is the *Salmonella* pathogenicity island 7 (SPI-7). Situated within SPI-7 is the *viaB* locus, an operon encoding regulatory (*tviA*), biosynthesis (*tviBCDE*), and export (*vexABCDE*) genes involved in the production of the virulence (Vi) capsular polysaccharide of *S.* Typhi [37] (Fig. S1A). The *viaB* locus has been shown to suppress Toll-like receptor (TLR) signaling pathways [13], [38], [39]. We therefore explored the contribution of the *viaB* locus on diminishing NF-κB activation in epithelial cells (Fig. 1D and S1B). Deletion of the entire *viaB* locus in *S.* Typhi (Δ*viaB* mutant; SW347) markedly increased the ability to activate NF-κB in epithelial HeLa cells (*P*<0.001). Akin to the findings with *S.* Typhimurium, NF-κB signaling induced by the *S.* Typhi *viaB* mutant was independent of invasiveness (Fig. S1C) but required a functional T3SS-1 since inactivation of *invA* in the *viaB* mutant background (Δ*viaB invA* mutant, STY4) completely abolished luciferase activity (*P*<0.001) (Fig. S1D). These results supported the idea that the *viaB* locus attenuates T3SS-1-induced, pro-inflammatory signaling pathways in human epithelial cells.

The *viaB* locus has been shown to alter interaction of *S.* Typhi with host cells through multiple distinct mechanisms (reviewed in [40]). The Vi capsular polysaccharide prevents complement deposition, phagocytosis, and TLR4 activation, while the regulatory protein TviA is known to dampen TLR5 signaling. We therefore wanted to discern whether the absence of NF-κB signaling in human epithelial cells is due to the production of the Vi capsule or due to altered gene expression mediated by TviA. To this end, the *tviA* gene cloned into a low copy number plasmid (pTVIA1) was introduced into a *S.* Typhi *viaB* mutant (STY2). Expression of *tviA* under control of the native promoter significantly lowered NF-κB activation (*P*<0.01) in comparison to cells infected with the *S.* Typhi *viaB* mutant carrying the empty vector control (pWSK29). Remarkably, expression of *tviA* reduced inflammatory responses to levels comparable to the *S.* Typhi wild-type strain (Fig. 1D and S1B), suggesting that the regulatory protein TviA is involved in dampening inflammatory responses in cultured human epithelial cells.

### TviA reduces T3SS-1-mediated inflammation in the bovine ligated ileal loop model

We had recently demonstrated that a *S.* Typhimurium strain carrying the *S.* Typhi *viaB* locus on a plasmid elicits less mucosal inflammation in a bovine ligated ileal loop model than the isogenic *S.* Typhimurium wild type ATCC14028 [38], raising the possibility that TviA might be involved in suppressing inflammatory responses *in vivo*. To delineate the relative contribution of the Vi capsule and the regulator TviA to reducing inflammatory responses in the bovine ligated ileal loop model [23], we repeated these studies with derivatives of *S.* Typhimurium strain ATCC 14028 in which the *phoN* gene in the chromosome had been replaced with the entire *S.* Typhi *viaB* locus (*phoN*::*viaB* mutant, TH170) or the *tviA* gene only (*phoN*::*tviA* mutant, SW474). In these strains, transcription of *tviA* and the downstream genes is solely controlled by the native *S.* Typhi promoter [41], [42]. This strategy was chosen to ensure that attenuation of intestinal inflammation in this model was not caused by introduction of the *viaB* locus on a multi-copy plasmid [38].

**Figure 2:**
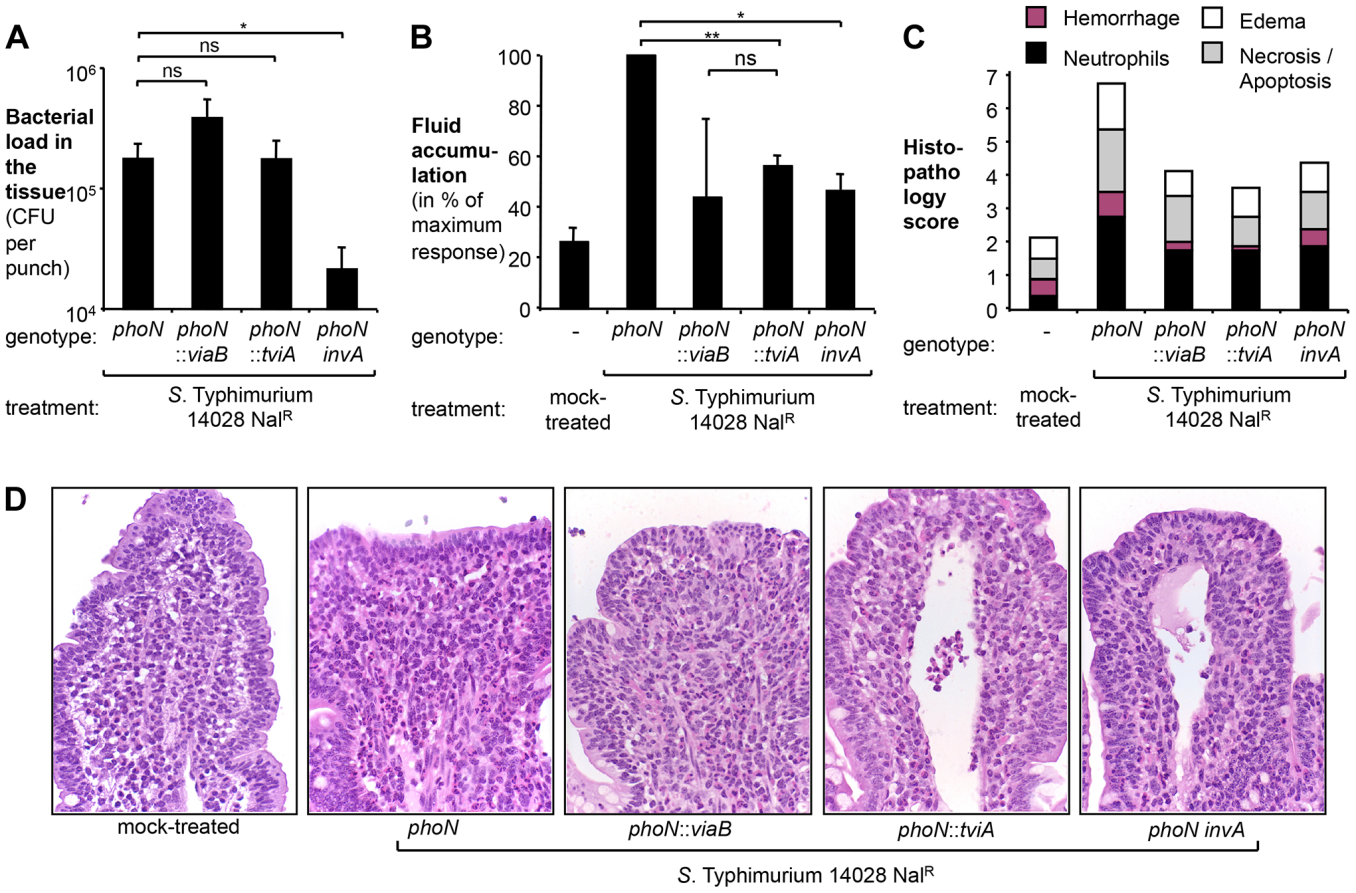
Expression of TviA in *S.* Typhimurium reduces early host responses. Bovine ligated ileal loops (N = 4 animals) were mock treated (LB broth) or infected with a *S.* Typhimurium ATCC14028 *phoN* mutant (AJB715), a *phoN*::*viaB* mutant (TH170), a *phoN*::*tviA* mutant (SW474), or a *phoN invA* mutant (SW737) pre-cultured in LB broth. Samples were collected 5 h after infection. (A) Bacterial load in the tissue was determined by treating tissue biopsies with Gentamicin and plating on selective media. Bars represent geometric means of bacterial loads ± standard error. (B) Fluid accumulation recorded 5 h after infection. Data is expressed as a percent of the response observed in loops infected with the *phoN* mutant (maximum response). Bars represent geometric means ± standard error. (C) Pathological changes in the ileal mucosa. Formalin-fixed ileal tissue was scored by the following criteria: hemorrhage (purple bars), infiltration with neutrophils (black bars), presence of edema (white bars), and necrosis/apoptosis (grey bars). Bars represent the average obtained from 4 animals. (D) Representative images of hematoxilin and eosin-stained sections of the ileal mucosa (magnification 60×). *, *P*<0.05; **, *P*<0.01; ns, not statistically significant.

We compared the *phoN*::*viaB* mutant and the *phoN*::*tviA* mutant to a strain carrying an antibiotic resistance gene inserted chromosomally in the *phoN* gene (*phoN* mutant, AJB715). The *phoN*::*viaB* mutant, the *phoN*::*tviA* mutant, and the isogenic *phoN* mutant were recovered in equal numbers from gentamycin-treated tissue samples five hours after inoculation (Fig. 2A), suggesting that neither the *tviA* gene nor the entire *viaB* locus interfered with tissue invasion. Consistent with our previous observations [38], the *phoN*::*viaB* mutant elicited less fluid accumulation (Fig. 2B) and less pathological changes in the mucosa (Fig. 2C and D) than the isogenic *phoN* mutant. Remarkably, expression of *tviA* alone (*phoN*::*tviA* mutant) significantly reduced fluid accumulation and inflammation compared to the *phoN* mutant (*P*<0.01). The responses elicited by the *phoN*::*tviA* mutant and the *phoN*::*viaB* mutant were indistinguishable, suggesting that the *viaB*-mediated attenuation of inflammatory responses five hours after inoculation of bovine ligated ileal loops with *S.* Typhimurium was mostly attributable to the action of the TviA regulatory protein. Taken together, these data suggested that gene regulation mediated by TviA could dampen inflammatory processes *in vivo*.

### TviA represses transcription of regulatory, structural, and effector proteins of the *S.* Typhi T3SS-1

A functional T3SS-1 is required for the induction of intestinal host responses in cattle [22], [24], [43]. A *S.* Typhimurium strain carrying a mutation in the T3SS-1 apparatus gene *invA* (*invA phoN* mutant, SW737) was significantly less invasive than a *phoN* mutant (Fig. 2A) (*P*<0.05). Interestingly, inactivation of *invA* (*invA phoN* mutant) reduced fluid accumulation (Fig. 2B) and intestinal inflammation (Fig. 2C and D) by a magnitude that was similar to that observed for the *phoN*::*tviA* mutant. This finding was consistent with the idea that TviA reduces T3SS-1-dependent host responses *in vivo*, prompting us to further investigate the mechanism by which TviA inhibits T3SS-1 gene expression.

TviA is a key activator of the *tviBCDEvexABCDE* operon but can also control transcription of genes outside its own operon (Fig. S2A). Expression of TviA results in diminished motility and flagellin secretion due to downregulation of the flagellar regulon by repressing transcription of the *flhDC* genes [42], [44]. FlhDC, the master regulator of flagellar gene expression, activates transcription of class II flagellar genes, such as *fliA* and *fliZ* [45], [46]. FliA is a positive regulator of class III flagellar genes, including flagellin [45], [47]. To determine whether reduced motility or diminished flagellin production could account for the TviA-dependent reduction in NF-κB activation, we inactivated the *fliC* gene encoding the sole flagellin of the monophasic serovar Typhi, thereby rendering strains carrying these mutations aflagellate and non-motile. Deletion of the entire *viaB* operon (Δ*viaB* Δ*fliC* mutant, SW483) in the *fliC* background (Δ*fliC* mutant, SW359) significantly increased NF-κB signaling in infected HeLa and HEK293 epithelial cells (Fig. S2B and S2C). Expression of TviA from a plasmid (pTVIA1) in a *viaB fliC* mutant reduced luciferase activity to levels comparable to the *fliC* mutant (Fig. S2B and S2C), demonstrating that TviA-dependent repression of NF-κB activation was flagellin-independent.

Gene expression profiling experiments suggest that TviA affects transcription of T3SS-1 genes through the following signaling cascade [42]: By repressing transcription of *flhDC*, TviA downregulates expression of FliZ. The regulatory protein FliZ is an activator of *hilA* [48]–[50], the master regulator of T3SS-1 genes [51], [52], thus placing T3SS-1 gene expression under negative control of TviA (Fig. S2A). We therefore analyzed the effect of TviA on the transcription of a subset of regulatory, structural, and effector proteins in *S.* Typhi (Fig. S3). Consistent with previous findings, deletion of the Vi capsule biosynthesis genes alone (Δ*tviB-vexE* mutant, SW74) did not alter transcription of T3SS-1 genes [42], [44]. In contrast, concomitant deletion of *tviA* and capsule biosynthesis genes (Δ*viaB* mutant, SW347) significantly enhanced transcription of the regulatory genes *flhD*, *hilA*, and *invF*, the structural component gene *prgH*, as well as the effector genes *sipA* and *sopE* (Fig. S3).

### In the absence of TviA, the *S.* Typhi T3SS-1 effector protein SopE is the major inducer of NF-κB activation in epithelial cells

We next determined which T3SS-1 effector proteins contributed to pro-inflammatory responses elicited by *S.* Typhimurium and *S.* Typhi. Previous work has demonstrated that SopE, SopE2, SopB, and SipA contribute to NF-κB activation in epithelial cells [15]–[17], [53]. The bacteriophage-encoded *sopE* gene is present in *S.* Typhi Ty2 but absent from *S.* Typhimurium strain ATCC 14028. To better model the contribution of TviA on attenuating T3SS-1-induced host responses, we chose to continue our studies using the *S.* Typhimurium strain SL1344, an isolate that carries the *sopE* gene. Consistent with previous reports [15]–[17], [53], we found that simultaneous inactivation of *sopE*, *sopE2*, *sopB*, and *sipA* (*sopE sopE2 sopB sipA* mutant, SW868) reduced the ability of the *S.* Typhimurium strain SL1344 to induce NF-κB activation to levels observed in an isogenic *S.* Typhimurium strain unable to translocate effector proteins (*invA* mutant; SW767) (Fig. S4). A *S.* Typhimurium strain only expressing SopE (*sopE2 sopB sipA* mutant, SW867) elicited considerable NF-κB activation. A moderate NF-κB activation was also observed with *S.* Typhimurium strains only expressing SopB (*sopE sopE2 sipA* mutant, SW972) or only expressing SipA (*sopE sopE2 sopB* mutant, SW940) (Fig. S4). Essentially no response was observed in cells infected with a SL1344 derivative that only expressed SopE2 (*sopE sopA sopB* mutant, SW973). Collectively, these data suggested that SopE was the most potent inducer of pro-inflammatory responses in this tissue culture model, while the contributions of SopB and SipA were more modest.

**Figure 3:**
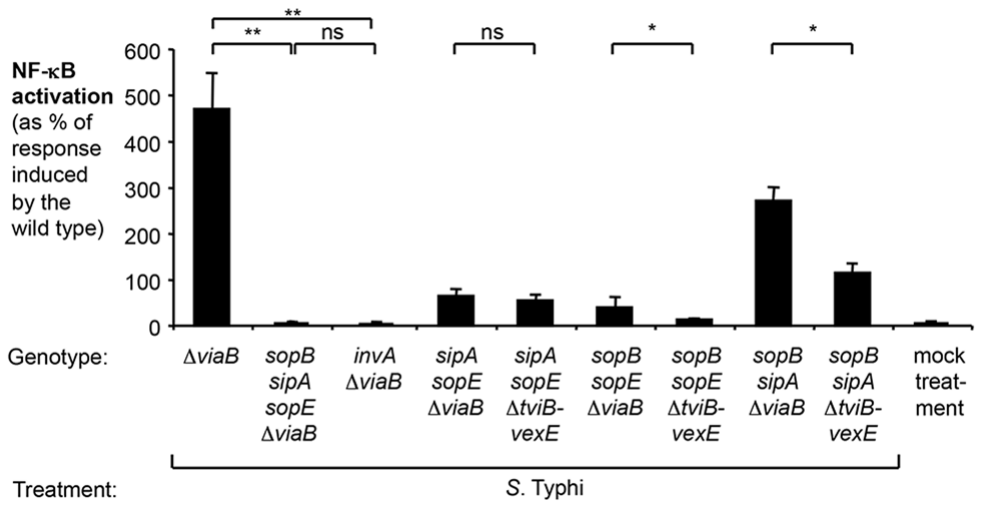
Effect of the regulator TviA on NF-κB activation triggered by *S.* Typhi T3SS-1 effectors. HeLa cells permanently transfected with a NF-κB-luciferase reporter system (HeLa 57A) were either mock treated (bacterial growth media alone) or infected with the *S.* Typhi wild type Ty2, a Δ*viaB* mutant (SW347), a *sopB sipA sopE* Δ*viaB* mutant (SW1217), an *invA* Δ*viaB* mutant (STY4), a *sipA sopE* Δ*viaB* mutant (SW1214), a *sipA sopE* Δ*tviB-vexE* mutant (SW1215), a *sopB sopE* Δ*viaB* mutant (SW1216), a *sopB sopE* Δ*tviB-vexE* mutant (SW1213), a *sopB sipA* Δ*viaB* mutant (SW1211), or a *sopB sipA* Δ*tviB-vexE* mutant (SW1212). After 5 h, luciferase activity was measured (N = 4). Bars represent geometric means ± standard error. *, *P*<0.05; **, *P*<0.01; ns, not statistically significant.

We next determined the potential contribution of the *S.* Typhi orthologues of these effectors to the induction of NF-κB signaling in the absence of the *tviA* gene (*S.* Typhi Δ*viaB* mutant, SW347) (Fig. 3). The *sopE2* gene is a pseudogene in *S.* Typhi Ty2 and was not further analyzed. Concomitant inactivation of *sopE*, *sipA* and *sopB* in the *S.* Typhi *viaB* mutant (*sopB sipA sopE* Δ*viaB* mutant, SW1217) completely abolished NF-κB-driven luciferase activity (Fig. 3). This indicated that, akin to the findings with the *S.* Typhimurium strain SL1344, SopE, SipA, and SopB are critical for the induction of inflammatory responses in epithelial cells upon infection with *S.* Typhi. A *S.* Typhi *viaB sopB sipA* mutant (SW1211) elicited pronounced NF-κB activation, but a more modest NF-κB activation was also observed with the *S.* Typhi *viaB sopE sipA* mutant (SW1214) and the *viaB sopE sopB* mutant (SW1216) (Fig. 3). These data suggested that SopE was the most potent inducer of pro-inflammatory responses in *S.* Typhi strains lacking the *tviA* gene while SopB and SipA contributed moderately. In contrast, diminished NF-κB activation was observed with *S.* Typhi *tviB-vexE* mutant (carrying the *tviA* gene) and its derivatives (Fig. 3). This intricate comparison between derivatives of the *viaB* mutant and the *tviB-vexE* mutant allowed us to preclude any confounding effects expression of the Vi antigen might have on gene regulation: both the *viaB* mutant and the *tviB-vexE* mutant are non-encapsulated and only differ in their capability of expressing *tviA*. In contrast, a simple *tviA* mutant would exhibit a pleiotropic effect, i.e. it would lack the regulatory TviA protein but at the same time exhibit virtually no production of the Vi antigen [37]. Collectively, these data suggested that TviA-mediated gene regulation reduced T3SS-1 effector-triggered NF-κB activation.

### TviA reduces activation of the Rac1 and NOD1/2 signaling pathway

**Figure 4:**
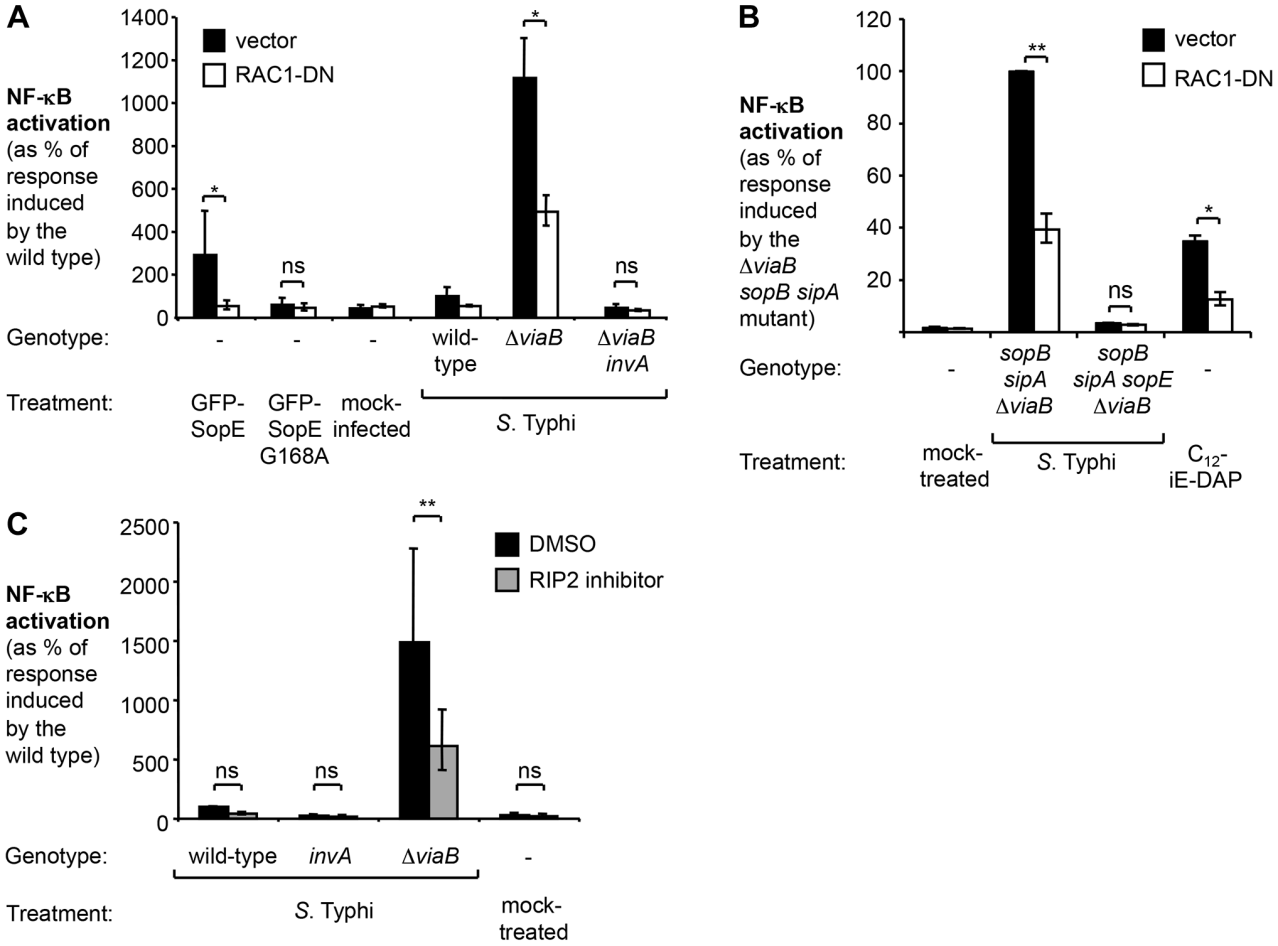
In the absence of *tviA*, *S.* Typhi induces NF-κB activation in a RAC1- and RIP2-dependent manner. (A and B) HeLa cells permanently transfected with a NF-κB luciferase reporter system (HeLa 57A) were transfected with pCMV-myc (vector, black bars) or pRAC1-DN (RAC1-DN, white bars) (A) Cells were transfected with the indicated GFP-SopE constructs or infected with the indicated *S.* Typhi strains for 5 h (N = 3). (B) Cells were mock-treated, infected with the indicated *S.* Typhi trains, or treated with the NOD1 agonist C_12_-iE-DAP (an acylated derivative of γ-D-Glu-mDAP) for 5 h. (C) HeLa 57A cells were either treated with dimethyl sulfoxide (DMSO) or RIP2 inhibitor SB203580 dissolved in DMSO and subsequently infected with the indicated *S.* Typhi strains or were mock treated (bacterial growth media alone) (N = 3). Bars represent geometric means ± standard error. *, *P*<0.05; **, *P*<0.01; ns, not statistically significant.

Since SopE triggered the most pronounced host responses in the absence of *tviA*, we focused our further analysis on this signaling pathway. Mechanistic studies in cultured epithelial cells have revealed that the bacterial guanine nucleotide exchange factor (GEF) SopE activates the Rho-family GTPase Ras-related C3 botulinum toxin substrate 1 (RAC1) [15]. Excessive stimulation of RAC1 by bacterial effectors is sensed by the nucleotide-binding oligomerization domain-containing protein 1 (NOD1) [30]. Activation of NOD1 leads to phosphorylation of the receptor-interacting serine/threonine-protein kinase 2 (RIP2) and activation of NF-κB signaling in epithelial cells [15], [20], [30], [54]. The NOD1/2 signaling pathway in HeLa cells can also be triggered by SipA [34], although this pathway plays a lesser role in the SopE-encoding strain SL1344 (Fig. S4). Taken together, these findings raised the possibility that TviA-mediated downregulation of SopE allows *S.* Typhi to abate immune recognition by the RAC1-NOD1/2-RIP2 signaling pathway. To test this hypothesis, we abrogated RAC1 and RIP2 signaling by either ectopically expressing a dominant negative form of RAC1 (RAC1-DN) [30], [55] or by treating cells with the RIP2 inhibitor (SB203580) (Fig. 4). Consistent with previous reports, ectopic expression of a GFP-SopE fusion protein alone was sufficient to induce NF-κB activation while no upregulation of this signaling pathway was observed with a GFP-SopE construct lacking GEF activity (GFP-SopE G168A) [30], [56]. Simultaneous expression of the GFP-SopE fusion protein and a RAC1-DN construct abrogated NF-κB signaling (Fig. 4A).

Infection of HeLa cells with the *S.* Typhi wild type or the T3SS-1-deficient *viaB invA* mutant did not result in a statistically significant increase in NF-κB activation and abrogation of RAC1 or RIP2 signaling did not further impact signaling (Fig. 4A, B, and C). In marked contrast, infection with the *S.* Typhi *viaB* mutant led to a substantial upregulation of NF-κB-driven responses. Abrogation of RAC1 or RIP2 activity significantly blunted the induction of NF-κB responses in cells infected with the *viaB* mutant. Moreover, NF-κB activation in cells infected with a *viaB sopB sipA* mutant was inhibited when cells were transfected with a plasmid construct encoding RAC1-DN (Fig. 4C), suggesting that SopE, translocated into host cells in the absence of TviA, could activate NF-κB signaling in a RAC1-dependent manner. Treatment with the RIP2 inhibitor did not impact T3SS-1-mediated invasion of *S.* Typhi strains towards epithelial cells (Fig. S5), excluding the possibility that the RIP2 inhibitor inadvertently interfered with the function of the T3SS-1 machinery. Collectively, these data supported the idea that TviA restricts activation of the RAC1-NOD1/2-RIP2 signaling pathway in *S.* Typhi-infected epithelial cells.

### Heterologous expression of TviA in *S.* Typhimurium blunts T3SS-1-dependent responses *in vivo*

**Figure 5:**
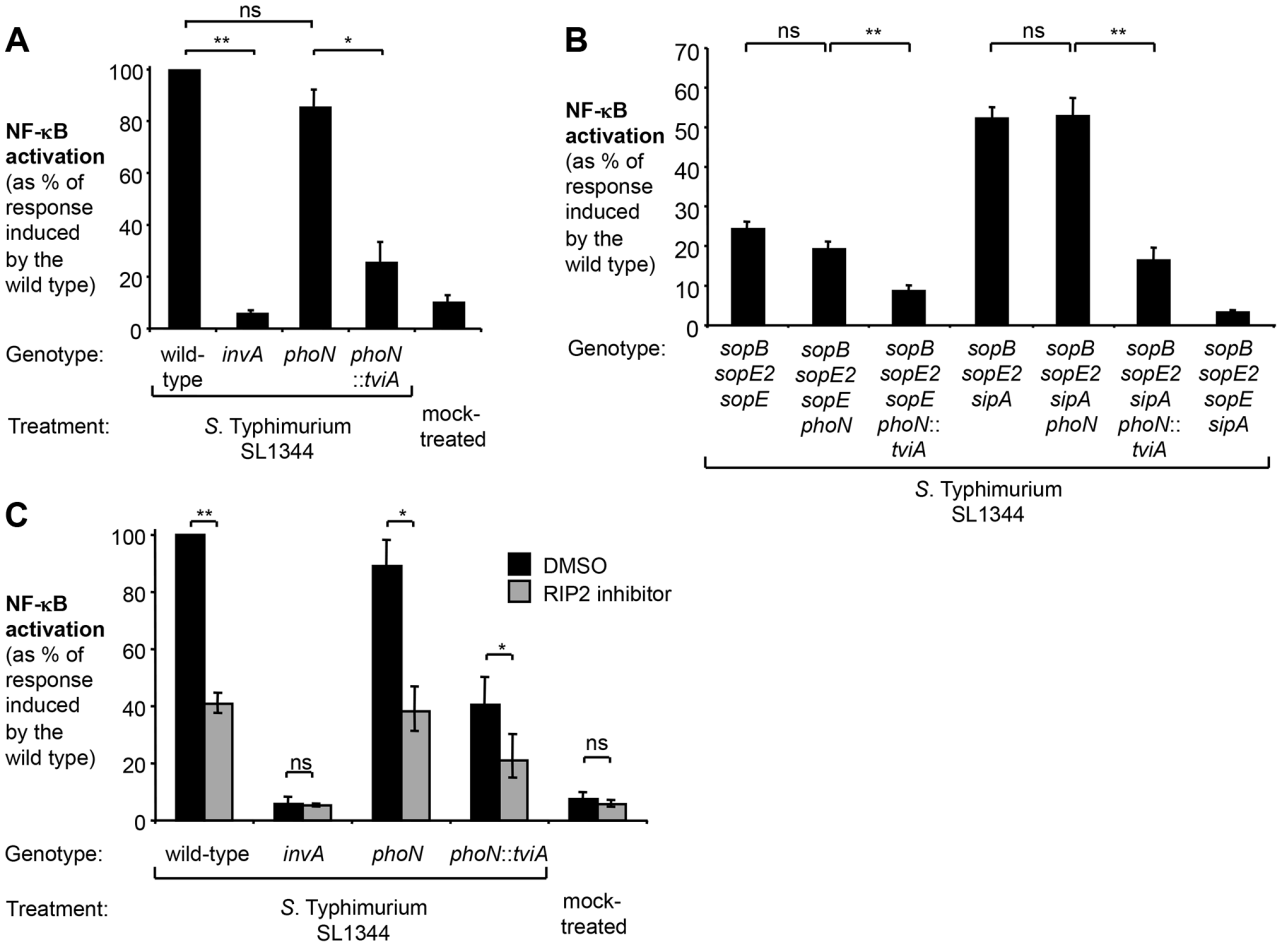
Expression of TviA in *S.* Typhimurium SL1344 reduces T3SS-1-driven NF-κB activation in epithelial cells. HeLa 57A cells were infected with *S.* Typhimurium or mock treated with bacterial growth media (mock treatment) and luciferase activity determined 5 h after infection (N = 4). (A) The SL1344 wild type, an *inv*A mutant (SW767), a *phoN* mutant (SW759), and a *phoN*::*tviA* mutant (SW760) were used to infect monolayers of HeLa 57A cells. (B) Cells were infected with the SL1344 wild type, an isogenic *sopB sopE2 sopE sipA* mutant (SW868), a *sopB sopE2 sopE* mutant (SW940), a *sopB sopE2 sipA* mutant (SW867), and derivatives thereof carrying a *phoN* (SW808; SW806) or a *phoN*::*tviA* (SW809; SW807) mutation, respectively. (C) Prior to infection with the indicated *S.* Typhimurium strains, cells were either treated with DMSO or the RIP2 inhibitor (SB203580) dissolved in DMSO. Bars represent geometric means ± standard error. *, *P*<0.05; **, *P*<0.01; ns, not statistically significant.

In addition to repressing T3SS-1 genes, TviA also suppresses flagella expression (Fig. S2A) [57]. Flagellin is known to induce pro-inflammatory responses by activating TLR5 [58] and the NLRC4- (nucleotide-binding oligomerization domain [NOD]-like receptor [NLR] family caspase-associated recruitment domain [CARD]-containing protein 4-) inflammasome [59], [60]. While our initial experiments in the bovine ligated ileal loop model suggest that TviA could mitigate mucosal inflammation (Fig. 2), it is conceivable that TviA-mediated gene regulation of flagellar biosynthesis could have affected flagellin-dependent innate immune pathways. To better study consequences of the expression of TviA on the RAC1-NOD1/2-RIP2 signaling pathway in an animal model, we therefore generated a *phoN*::*tviA* mutant in the *S.* Typhimurium SL1344 background (SW760). Akin to the findings with *S.* Typhi, expression of TviA in *S.* Typhimurium reduced transcription of T3SS-1 genes (Fig. S3) and the *phoN*::*tviA* mutant elicited significantly less (P<0.05) NF-κB activation than the *phoN* control strain (Fig. 5A and S6). We next introduced the *tviA* gene into SL1344 derivatives that only expressed the most potent inducers of the NF-κB pathway, SipA (*sopE sopE2 sopB phoN*::*tviA* mutant; SW809) and SopE (*sopE2 sopB sipA phoN*::*tviA* mutant; SW807). Upon infection of HeLa cells (Fig. 5B), strains carrying the *phoN*::*tviA* insertion elicited significantly less luciferase activity than the respective *phoN* mutants (P<0.01), indicating that TviA is able to reduce the NF-κB activation elicited by the *S.* Typhimurium orthologues of SopE and SipA. Inhibition of RIP2 significantly reduced NF-κB activation levels induced by the wild-type strain or the *phoN* mutant (Fig. 5C). The modest response induced by the *phoN*::*tviA* mutant was further blunted by inhibition of RIP2 signaling (*P*<0.05) (Fig. 5C), suggesting that TviA-mediated regulation of T3SS-1 is partially able to avoid induction of the NOD1/2-RIP2 pathway *in vitro*.

**Figure 6:**
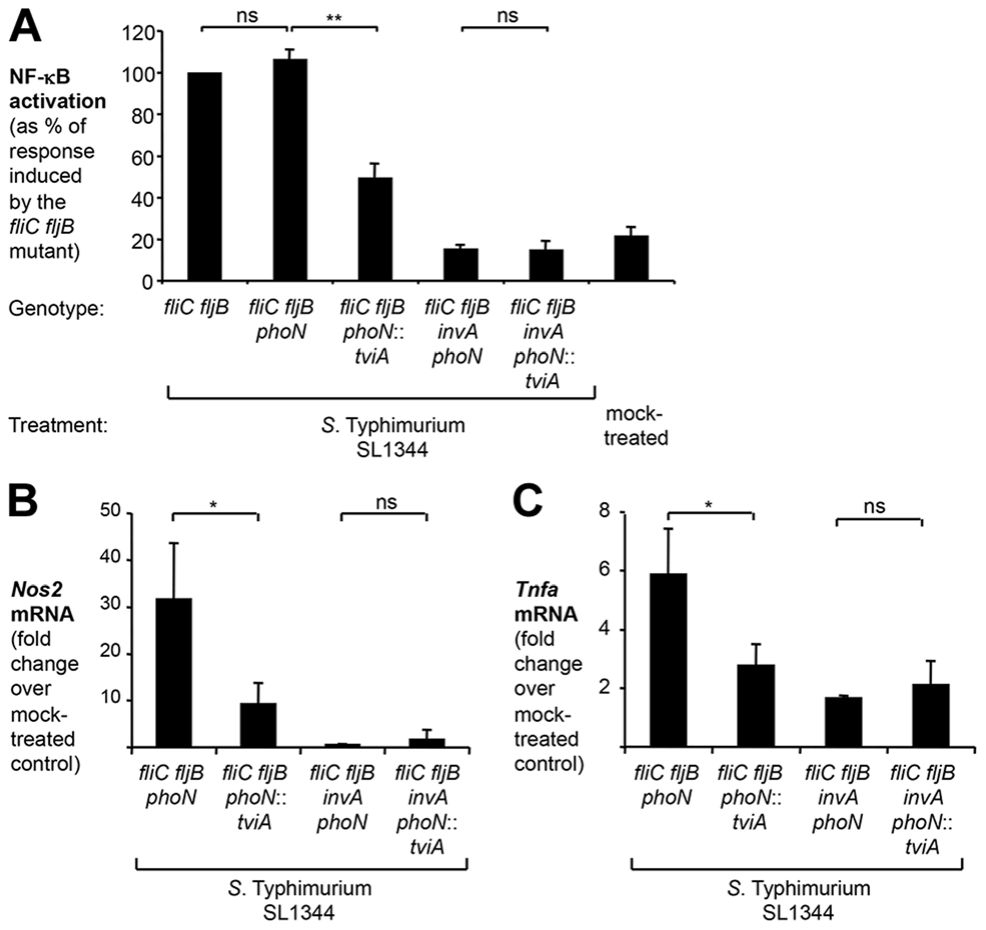
TviA reduces T3SS-1-dependent, flagellin-independent inflammatory responses in the cecal mucosa. (A) HeLa 57A cells were infected with an aflagellate SL1344 *fliC fljB* mutant (SW762), a *fliC fljB phoN* mutant (SW793), a *fliC fljB phoN*::*tviA* mutant (SW764) and derivatives carrying an additional mutation in *invA* (SW794; SW766). NF-κB activation was determined as described above. (B and C) Groups of streptomycin-pretreated C57BL/6 mice were intragastrically inoculated with either a *S.* Typhimurium SL1344 *fliC fljB phoN* mutant (N = 8), a *fliC fljB phoN*::*tviA* mutant (N = 8), a *fliC fljB phoN invA* mutant (N = 4), a *fliC fljB phoN*::*tviA invA* mutant (N = 4), or LB broth (mock-treated; N = 3). 12 h after infection, the relative abundance of *Nos2* (B) and *Tnfa* (C) mRNA was determined by qRT-PCR. Bars represent geometric means ± standard error. *, *P*<0.05; **, *P*<0.01; ns, not statistically significant.

To exclude any effects of TviA on flagellin-dependent pathways, we introduced the *phoN*::*tviA* mutation into a non-motile *S.* Typhimurium strain lacking phase 1 and 2 flagellins, FliC and FljB (*fliC fljB* mutant, SW762) (Fig. 6A). Both the *fliC fljB* mutant and the *fliC fljB phoN* mutant (SW793) elicited significant levels of NF-κB activation in cultured epithelial cells (Fig. 6A), while this response was greatly reduced in cells infected with the *fliC fljB phoN*::*tviA* mutant (SW764). Inactivation of the essential T3SS-1 gene *invA* completely abolished the ability to induce NF-κB signaling (Fig. 6A).

To directly assess the ability of TviA to impede inflammatory processes in the intestinal mucosa, we used the Streptomycin pre-treated mouse model [61]. In this model, detection of cytosolic access by the *S.* Typhimurium T3SS-1 through the NOD1/2 signaling pathway contributes to intestinal inflammation early during infection [30], [34], [62]. Compared to mock infected mice, transcript levels of the pro-inflammatory genes *Nos2*, encoding inducible nitric oxide synthase (iNOS), and *Tnfa*, encoding tumor necrosis factor (TNF)-α, were significantly (*P*<0.05) elevated in the cecal mucosa at 12 hours after infection with a non-flagellated *S.* Typhimurium *phoN fliC fljB* mutant (SW793) (Fig. 6B and C). Introduction of the *S.* Typhi *tviA* gene into a *S.* Typhimurium *fliC fljB* mutant (*phoN*::*tviA fliC fljB* mutant, SW764) significantly (*P*<0.05) reduced pro-inflammatory gene expression (Fig. 6B and 6C), but not bacterial numbers recovered from intestinal contents or Peyer's patches (Fig. S7). Inflammatory responses observed in the cecal mucosa at this early time point were T3SS-1-dependent, because introduction of a mutation in *invA* abrogated the ability of *S.* Typhimurium to elicit pro-inflammatory gene expression. Collectively, these data suggested that TviA represses T3SS-1-dependent, early inflammatory responses *in vivo* through a flagellin-independent mechanism.

## Discussion

*S.* Typhi invades the intestinal mucosa without triggering the massive neutrophil influx observed during gastroenteritis caused by non-typhoidal serovars. Here we show that one mechanism for attenuating host responses is a TviA-mediated repression of T3SS-1, a virulence factor known to induce potent inflammatory host responses. Effector molecules translocated by the T3SS-1 into the host cell cytosol activate Rho-family GTPases [15]–[17]. The activation of Rho-family GTPases is a pathogen-induced process that is sensed by NOD1 [21], [30], which ultimately results in the activation of pro-inflammatory responses *in vitro* [15], [20], [54] and *in vivo* [30], [62]. However, *S.* Typhi requires a functional T3SS-1 to invade the intestinal epithelium during infection [27]. Our data suggest that S. Typhi might have evolved to invade the intestinal epithelium without inducing a potent antibacterial inflammatory response by regulating T3SS-1 expression in a TviA-dependent manner. Osmoregulation prevents expression of TviA in the intestinal lumen, which renders *S.* Typhi invasive [42] (Fig. 2A). However, TviA expression is rapidly upregulated upon entry into tissue [63], resulting in repression of T3SS-1 and flagella expression while biosynthesis of the Vi capsule is induced [42], [44]. Here we show that TviA prevented NF-κB activation in epithelial cells by reducing T3SS-1-dependent activation of RAC1. Furthermore, the TviA-mediated reduction of T3SS-1-dependent inflammatory responses elicited at early time points in animal models was independent of flagella and the Vi capsule. These data support the hypothesis that TviA attenuates inflammation because it rapidly turns off T3SS-1 expression upon entry into tissue, thereby concealing a pathogen-induced process from the host.

Bovine ligated ileal loops are suited to model the initial 12 hours of host pathogen interaction, a time period during which inflammatory responses are largely T3SS-1-dependent [22], [23], [64]. Similarly, in the mouse colitis model, inflammatory responses elicited in the cecum at early time points (i.e. during the first 2 days) after infection are largely T3SS-1-dependent [61], [65], [66]. However, mechanisms independent of T3SS-1 are responsible for cecal inflammation observed at later time points (i.e. at days 4 and 5 after infection) in the mouse colitis model [65]. Expression of the *S.* Typhi Vi capsular polysaccharide in *S.* Typhimurium leads to an attenuation of these T3SS-1-independent inflammatory responses in the mouse colitis model [41], by reducing complement activation and TLR4 signaling [67], [68]. Thus the *viaB* locus reduces intestinal inflammation by multiple different mechanisms (Fig. S2A). A TviA-mediated repression of T3SS-1 reduces early inflammatory responses while the Vi capsular polysaccharide attenuates responses generated through T3SS-1-independent mechanisms at later time points. It is tempting to speculate that the result of these immune evasion mechanisms is a reduction in the intestinal inflammatory response that could contribute to differences in disease symptoms caused by typhoidal and non-typhoidal serotypes.

## Materials and Methods

### Ethics statement

This study was performed in strict accordance with the recommendations in the Guide for the Care and Use of Laboratory Animals of the National Institutes of Health. The protocol on mouse experiments was approved by the Institutional Animal Care and Use Committee of the University of California, Davis (Permit Number: 16179). The protocol on calf experiments was approved by the Institutional Committee at the Universidade Federal de Minas Gerais, Brazil (Permit Number: CETEA 197/2008).

### Bacterial strains and culture conditions

The bacterial strains, including relevant properties, are listed in table 1. Unless noted otherwise, bacteria were aerobically grown at 37°C in Luria-Bertani (LB) broth (10 g/l tryptone, 5 g/l yeast extract, 10 g/l NaCl) or LB agar (15 g/l agar). To induce expression of *tviA* and Vi capsule biosynthesis genes, an overnight culture in LB broth was diluted 1∶50 in tryptone yeast extract (TYE) broth (10 g/l tryptone, 5 g/l yeast extract) or Dulbecco's modified Eagle's medium (DMEM) as indicated and incubated aerobically at 37°C for 3 h. When appropriate, antibiotics were added to LB broth cultures or LB agar plates at the following concentrations: carbenicillin (0.1 mg/ml), chloramphenicol (0.03 mg/ml), kanamycin (0.05 mg/ml), nalidixic acid (0.05 mg/ml), and tetracycline (0.01 mg/ml).

**Table 1:** Bacterial strains and plasmids used in this study.

Strain designation	Relevant characteristics^a^/Genotype	Source/Reference
*S.* Typhi		
Ty2	Wild-type strain, Vi^+^	ATCC 700931^b^
STY2	Ty2 Δ*viaB*::Kan^R^	[13]
STY4	Ty2 Δ*viaB*::Kan^R^ *invA*::pINV5 (Cm^R^)	[13]
SW74	Ty2 Δ*tviB-vexE*::Cm^R^, Vi^−^	[57]
SW222	Ty2 *invA*::pINV5 (Cm^R^)	[57]
SW347	Ty2 Δ*viaB*, Vi^−^	[42]
SW359	Ty2 Δ*fliC*	[42]
SW398	Ty2 Δ*viaB* Δ*fliC invA*::pSW127	This study
SW483	Ty2 Δ*viaB* Δ*fliC*	[42]
SW611	Ty2 Δ*tviB-vexE*::Cm^R^ *invA*::pSW127	This study
SW904	Ty2 Δ*tviB-vexE*, Vi^−^	This study
SW1207	Ty2 Δ*viaB sopB*::Mu*d*J	This study
SW1208	Ty2 *tviB-vexE sopB*::Mu*d*J	This study
SW1209	Ty2 Δ*viaB* Δ*sopE*	This study
SW1210	Ty2 *tviB-vexE* Δ*sopE*	This study
SW1211	Ty2 Δ*viaB sopB*::Mu*d*J Δ*sipA*	This study
SW1212	Ty2 Δ*tviB-vexE sopB*::Mu*d*J Δ*sipA*	This study
SW1213	Ty2 Δ*tviB-vexE sopB*::Mu*d*J Δ*sopE*	This study
SW1214	Ty2 Δ*viaB* Δ*sopE*Δ*sipA*	This study
SW1215	Ty2 Δ*tviB-vexE* Δ*sopE*Δ*sipA*	This study
SW1216	Ty2 Δ*viaB sopB*::Mu*d*J Δ*sopE*	This study
SW1217	Ty2 Δ*viaB sopB*::Mu*d*J Δ*sipA* Δ*sopE*	This study
*S.* Typhimurium		
AJB715	IR715 *phoN*::Kan^R^	[73]
CS019	ATCC14028 *phoN*::Tn*10d*Cm	[74]
IR715	ATCC14028 Nal^R^	[75]
SL1344	Strep^R^	[76]
SPN305	IR715 Δ*fliC*::pSPN29	[42]
SW284	IR715 *phoN*: Cm^R^	[42]
SW399	IR715 *invA*::pSW127	[71]
SW474	IR715 *phoN*::*tviA*-Cm^R^	[42]
SW562	IR715 Δ*invA*::Tet^R^	[77]
SW737	IR715 *phoN*::Kan^R^ Δ*invA*::Tet^R^	This study
SW751	IR715 *phoN*::pSW208	This study
SW759	SL1344 *phoN*::Cm^R^	This study
SW760	SL1344 *phoN*::*tviA*-Cm^R^	This study
SW761	SL1344 Δ*fliC*	This study
SW762	SL1344 Δ*fliC fljB5001*::Mu*d*J	This study
SW764	SL1344 Δ*fliC fljB5001*::Mu*d*J *phoN*::*tviA*-Cm^R^	This study
SW766	SL1344 Δ*fliC fljB5001*::Mu*d*J *phoN*::*tviA*-Cm^R^ Δ*invA*::Tet^R^	This study
SW767	SL1344 Δ*invA*::Tet^R^	This study
SW793	SL1344 Δ*fliC fljB5001*::Mu*d*J *phoN*::pSW208	This study
SW794	SL1344 Δ*fliC fljB5001*::Mu*d*J *phoN*::pSW208 Δ*invA*::Tet^R^	This study
SW798	SL1344 *sopB*::Mu*d*J	[30]
SW800	SL1344 *sopE2*::pSB1039	[30]
SW806	SL1344 Δ*sipA sopB*::Mu*d*J *sopE2*::pSB1039 *phoN*::Tn*10d*Cm	This study
SW807	SL1344 Δ*sipA sopB*::Mu*d*J *sopE2*::pSB1039 *phoN*::*tviA*-Cm^R^	This study
SW808	SL1344 Δ*sopE sopB*::Mu*d*J *sopE2*::pSB1039 *phoN*::Tn*10d*Cm	This study
SW809	SL1344 Δ*sopE sopB*::Mu*d*J *sopE2*::pSB1039 *phoN*::*tviA*-Cm^R^	This study
SW839	SL1344 Δ*sipA*::pSW244	This study
SW867	SL1344 Δ*sipA sopB*::Mu*d*J *sopE2*::pSB1039	[30]
SW868	SL1344 Δ*sipA*Δ*sopE sopB*::Mu*d*J *sopE2*::pSB1039	[30]
SW940	SL1344 Δ*sopE sopB*::Mu*d*J *sopE2*::pSB1039	This study
SW972	SL1344Δ*sipA* Δ*sopE sopE2*::pSB1039	This study
SW973	SL1344 Δ*sipA* Δ*sopE sopB*::Mu*d*J	[30]
SW974	IR715 Δ*sipA*::pSW244	[30]
SW976	SL1344 Δ*sopE*	[78]
SW977	SL1344 Δ*sopE*::pSW245	This study
SW1009	SL1344Δ*sipA* Δ*sopE*	[30]
TH170	IR715 *phoN*::*viaB*	[41]
*E. coli*		
TOP10	F^−^ *mcr*A Δ(*mrr*-*hsdRMS-mcrBC*) Φ*80lacZ*Δ*M15* Δ*lacX*74 *recA*1 *araD*139 Δ(*ara leu*) 7697 *galU galK rpsL* (Strep^R^) *endA1 nupG*	Life Technologies
DH5α λ*pir*	F^−^ *endA1 hsdR17* (r^−^m^+^) *supE44 thi-1 recA1 gyrA relA1* Δ(*lacZYA-argF*)*U169* Φ*80lacZ* Δ *M15* λ*pir*	Laboratory strain collection
S17-1 λ*pir*	*recA1 thi pro hsdR* (r^−^m^+^) *zxx*::RP4 2-(Tet^R^::Mu) (Kan^R^::Tn7) λ*pir*	[79]

a Cm^R^: Chloramphenicol resistance; Kan^R^: Kanamycin resistance; Nal^R^: Nalidixic acid resistance; Strep^R^: Streptomycin resistance; Tet^R^: Tetracycline resistance (*tetRA*).

b American Type Culture Collection, Manassas, VA.

### Construction of plasmids

Standard cloning techniques were performed to generate the plasmids listed in table 2. Cloning vectors and *ori*(R6K)-based suicide plasmids were routinely maintained in *E. coli* TOP10 and DH5α λ*pir*, respectively.

**Table 2:** Plasmids used in this study.

Plasmid designation	Relevant characteristics^a^/Genotype	Source/Reference
pCMV-myc	*ori*(pMB1) *bla P*_CMVIE_ myc-tag	Clontech
pCR2.1	Cloning vector	Life Technologies
pEP185.2	*ori*(R6K) *mobRP4 cat*	[80]
pEGFP-C1	*ori*(pMB1) Kan^R^ *P*_CMVIE_ EGFP	Clontech
pGFP-SopE	*sopE* cloned into pEGFP-C1; *N*-terminal GFP tag	[30]
pGFP-SopE-G168A	G168A amino acid substitution in SopE in pEGFP-C1; *N*-terminal GFP tag	[30]
pNFkB-luc	NF-κB -responsive luciferase reporter plasmid	[81]
pRAC1-DN	Dominant-negative form of h*RAC1* cloned into pCMV-myc; T17N amino acid substitution; *N*-terminal myc tag.	[30]
pRDH10	*ori*(R6K) *mobRP4 cat tetC sacRB*	[82]
pRI203	*Y. pseudotuberculosis* invasin gene in pREG153	[83]
pSW28	Upstream and downstream regions of the *tviBCDEvexABCDE* region of *S.* Typhi Ty2 in pGP704	[57]
pSW208	Internal fragment of the *S.* Typhimurium *phoN* gene cloned into pEP185.2	This study
pSW233	Upstream and downstream regions of the *tviBCDEvexABCDE* region of *S.* Typhi Ty2 in pRDH10	This study
pSW245	Upstream and downstream region of the *S.* Typhimurium *sopE* gene in pRDH10	[78]
pTK-LacZ	Normalization of transfection efficiency	[84]
pTVIA1	*tviA* under control of its native promoter in pWSK29	[38]
pWSK29	*ori*(pSC101) *bla*	[85]

An internal fragment of the *phoN* coding sequence was PCR amplified from the *S.* Typhimurium IR715 chromosome using the primers listed in table 3, subcloned into pCR2.1 (TOPO TA cloning kit, Life Technologies), and cloned into pEP185.2 utilizing the unique XbaI and SacI restriction sites to give rise to pSW208. To generate pSW233, pSW28 was digested with EcoRI and the DNA fragment comprising the joint upstream- and downstream regions of the *tviB* and *vexE* genes, respectively, was cloned into the EcoRI site of pRDH10.

**Table 3:** Primers used in this study.

Target	Sequence^c^	Reference
Mutagenesis		
*S.* Typhimurium *phoN*	5′-TCTAGACGATGGAAACAAGCTGC-3′	This study
	5′-GAGCTCTACTAATGCCAGAAGTGT-3′	
Real time PCR		
*Salmonella gmk*	5′-TTGGCAGGGAGGCGTTT-3′	[86]
	5′-GCGCGAAGTGCCGTAGTAAT-3′	
*Salmonella flhD*	5′-ACAGCGTTTGATCGTCCAG-3′	[63]
	5′-GTTTGCCATCTCTTCGTTGA-3′	
*Salmonella hilA*	5′-ATTAAGGCGACAGAGCTGGA-3′	[42]
	5′-GAATAGCAAACTCCCGACGA-3′	
*Salmonella invF*	5′-GTTGTCGCACCAGTATCAGG-3′	This study
	5′-TCGGATTCAGCATATGTCGT-3′	
*Salmonella prgH*	5′-CACTGAACGGCTGTGAGTTT-3′	This study
	5′-CGGCAGGTATATCAGGGAGT-3′	
*Salmonella sipA*	5′-TTCAAATAATGTCGCCGGTA-3′	This study
	5′-TTCATCAGTAGCGTCTTCGC-3′	
*Salmonella sopE*	5′-CAACACACTTTCACCGAGGA-3′	This study
	5′-ATCATTGAGCGTTTGAAGCA-3′	
Murine *Gapdh*	5′-TGTAGACCATGTAGTTGAGGTCA-3′	[87]
	5′-AGGTCGGTGTGAACGGATTTG-3′	
Murine *Tnfa*	5′-AGCCAGGAGGGAGAACAGAAAC-3′	[68]
	5′-CCAGTGAGTGAAAGGGACAGAACC-3′	
Murine *Nos2*	5′-TTGGGTCTTGTTCACTCCACGG-3′	[88]
	5′-CCTCTTTCAGGTCACTTTGGTAGG-3′	

c restriction endonuclease cleavage sites are underlined.

### Generation of mutants by allelic exchange

Plasmids were introduced into S17-1 λ*pir* and conjugation performed as described previously [57]. The unmarked *S.* Typhi Δ*tviB-vexE* mutant SW904 was constructed by inserting the plasmid pSW233 into the STY2 mutant chromosome, selecting for single crossover events (creating merodiploids) on LB agar plates containing Cm and Kan. Sucrose selection was performed as described previously [69] to select for a second crossover event, thus effectively deleting the *tviBCDEvexABCDE* genes, yielding SW904. The deletion was confirmed by PCR. To facilitate transduction of the unmarked Δ*sopE* mutation, pSW245 was introduced in this locus in the SW976 chromosome by conjugation with S17-1 λ*pir* as the donor strain, creating SW977 as an intermediate.

### Construction of mutants by P22-mediated generalized phage transduction

Phage P22 HT *int-105* was utilized for generalized phage transduction in *S.* Typhimurium as described previously [70]. For *S.* Typhi recipients, a similar protocol was followed except the multiplicity of infection (MOI) was increased to 100.

A phage lysate of SW399 was used to transduce the *invA*::pSW127 mutation into SW483 and SW74, thus generating the *S.* Typhi Δ*viaB* Δ*fliC invA* mutant (SW398) and the Δ*tviB-vexE invA* mutant (SW611). SW1207 and SW1208 were created by transducing the *sopB::MudJ* mutation from SW798 into the Δ*viaB* mutant (SW347) and the Δ*tviB-vexE* mutant (SW904), respectively. The *S.* Typhi Δ*viaB* Δ*sopE* (SW1209), Δ*tviB-vexE* Δ*sopE* (SW1210), Δ*viaB sopB*::Mu*d*J Δ*sopE* (SW1216), and Δ*tviB-vexE sopB*::Mu*d*J Δ*sopE* (SW1213) mutants were constructed by transducing the Δ*sopE*::pSW245 mutation from SW977 into SW347, SW904, SW1207, and SW1208, respectively. Subsequent sucrose selection allowed selecting for mutants that had lost the plasmid by allelic exchange and generated a clean Δ*sopE* mutation, thus creating SW1209, SW1211, SW1216, and SW1213, respectively. Similarly, a P22 lysate of SW839 was used to transduce the Δ*sipA*::pSW244 mutation (SW839) into SW1207, SW1208, SW1209, and SW1210. The intermediates were subjected to sucrose selection, thus creating the clean Δ*sipA* mutation of strains SW1211, SW1212, SW1214, and SW1215, respectively. The Δ*viaB sopB*::Mu*d*J Δ*sipA* Δ*sopE* mutant (SW1217) was generated through transduction of the Δ*sopE*::pSW245 mutation from SW977 into the SW1211 chromosome and sucrose selection.

The *S.* Typhimurium SL1344 derivatives SW759 and SW760 were established by transducing the *phoN*::Cm^R^ and *phoN*::*tviA*-Cm^R^ mutations from SW284 and SW474 into the SL1344 wild type. Transduction of the Δ*fliC*::pSPN29 from SPN305 into the SL1344 wild type and subsequent sucrose selection gave rise to the SL1344 *fliC* deletion mutant SW761. Subsequent introduction of the *fljB5001*::Mu*d*J into this strain led to the SL1344 Δ*fliC fljB5001*::Mu*d*J mutant (SW762). To construct SW764 and SW793, the *phoN*::*tviA*-Cm^R^ (SW474) and *phoN*::pSW208 (SW751) mutations were transduced separately into SW762. Invasion-deficient derivatives of these strains were generated by transducing the *invA*::Tet^R^ mutation from SW562 into SW764, SW793, and SL1344, thus creating strains SW766, SW794, and SW767, respectively. SW806, SW807, SW808, and SW809 were generated by transducing the *phoN*::Tn*10d*Cm (CS019) or *phoN*::*tviA*-Cm^R^ (SW474) into SW867 or SW940. The Δ*sipA*::pSW244 mutation (SW974) was moved into the SL1344 wild type to create SW839. SW940 was established by transduction of the *sopB*::Mu*d*J mutation (SW798) into SW976 and subsequent introduction of the *sopE2*::pSB1039 mutation (SW800). A P22 phage lysate of SW800 was used to create SW972 using SW1009 as the recipient strain. The *phoN*::Kan^R^ mutation from AJB715 was transduced into SW562 to give rise to the *phoN*::Kan^R^ *invA*::Tet^R^ mutant (SW737).

### Tissue culture experiments

HeLa 57A cells [29], [34] were generously provided by R. T. Hay (the Wellcome Trust Centre for Gene Regulation and Expression, College of Life Sciences, University of Dundee, United Kingdom). HEK-293 cells were obtained from ATCC (ATCC CRL-1573). Both cells lines were routinely cultured at 37°C in a 5% CO_2_ atmosphere in DMEM containing 10% fetal bovine serum (FBS) (Life Technologies). For NF-κB activation and invasion experiments, cells were seeded in 24-well plates and 48-well plates (Corning) at densities of 1×10^5^ cells/well and 2×10^5^ cells/well, respectively, and incubated for 24 h prior to subsequent experiments.

### Measurement of NF-κB activation in epithelial cells

*S.* Typhi and S. Typhimurium strains were pre-cultured in TYE broth as described above. HeLa 57A cells or HEK-293 cells transfected with a NF-κB -luciferase reporter construct were infected with the indicated strains at a final concentration of approximately 10^6^ colony forming units (CFU)/ml. To synchronize the infection, plates were centrifuged for 5 min at 500 g at room temperature. After 3 h, cells were washed with DPBS and incubated at 37°C for an additional 2 h in the presence of DMEM containing 10% FBS. Cells were washed in DPBS, lysed in 0.1 ml of reporter lysis buffer (Promega), and firefly luciferase activity was measured using the luciferase assay system (Promega) in a FilterMax3 microplate reader (Molecular Devices). Results are expressed as percentage of maximum signal elicited in each individual assay. In some experiments, cells were treated 30 min prior to infection until the end of the experiment with either DMSO (vehicle control) or the RIP2-inhibitor SB203580 at a final concentration of 10 µM dissolved DMSO. The NOD1 agonist C_12_-iE-DAP (Invivogen) was added a final concentration of 100 ng/ml.

For transfection assays [34], HeLa 57A cells were grown to a confluency of about 60% and transiently transfected with a total of 250 ng of plasmid DNA, consisting of 50 ng of the β-galactosidase-encoding vector pTK-LacZ, and either 200 ng of pCMV-myc (control vector) or 100 ng pRAC1-DN and 100 ng of control vector. For co-transfection with pGFP-SopE constructs, 50 ng of pTK-LacZ, 10 ng of the pGFP-SopE plasmid, 90 ng of pEGFP (empty vector), and 100 ng of either pCMV-myc or pRAC1-DN was added. HEK-293 cells were transfected with 25 ng of pTK-LacZ and 25 ng of pNFkB-luc. 48 h after transfection, cells were infected with the indicated *Salmonella* strains or mock-treated (LB broth) as described above. Efficiency of transfection was normalized by adjusting luciferase values to β-galactosidase values.

### Invasion assays

Invasiveness of the indicated *Salmonella* strains was determined using a Gentamicin protection assay as described previously [71]. Briefly, HeLa 57A cells were infected at a MOI of 5 with *Salmonella* strains pre-cultured in TYE broth. After 1 h, cells were washed and media containing 0.1 mg/ml Gentamicin was added for 90 min. Diluted cell lysates (0.5% Triton-X-100) were spread on LB agar plates to determine the number of CFU per well. Invasiveness was calculated as percentage of recovered bacteria compared to the inoculum.

### Bacterial gene expression analysis

Overnight cultures of the indicated *S.* Typhi and *S.* Typhimurium strains were diluted 1∶50 in TYE broth and incubated at 37°C for 3 h. Total RNA was extracted from approximately 2×10^9^ CFU using the Aurum Total RNA Mini Kit (Biorad). 1 µg of total RNA was subjected to an additional DNase treatment (DNA-free kit, Life Technologies) and converted to cDNA using MuLV reverse transcriptase (Life Technologies) in a 25 µl volume as described previously [71]. 4 µl of this cDNA was used as the template for real time PCR analysis with the primers listed in table 3. Data was acquired on a ViiA 7 real-time PCR instrument (Life Technologies). Relative target gene expression was normalized to mRNA levels of the house keeping gene *gmk*, encoding guanylate kinase (ΔΔ*Ct* method). DNA contamination was less than 1% for all amplicons as determined by a separate RT-PCR mock reaction lacking reverse transcriptase.

### Bovine ligated ileal loop model

*Salmonella* Typhimurium was cultured in LB broth at 37°C under agitation, followed by subculture in fresh LB (without antibiotics) for 3 hours, at 37°C under agitation. Four 3–4 week-old male healthy *Salmonella*-free Holstein calves were used in this study. Ligated ileal loops were surgically prepared as previously described [23]. Ligated loops were mock treated with intraluminal injection of sterile LB broth or inoculated with 3 ml of suspensions containing 1×10^8^ CFU of the *S.* Typhimurium ATCC14028 *phoN* mutant (AJB715), a *phoN::viaB* mutant (TH170), a *phoN::tviA* mutant (SW474), or a *phoN invA* mutant (SW737). Ligated loops were surgically removed at 5 h after infection for tissue sampling and measurement of intraluminal fluid accumulation. Samples containing the intestinal mucosa and the associated lymphoid tissue were collected with a 6 mm biopsy punch. Each intestinal biopsy was kept in sterile PBS with 50 µg/ml of gentamicin for 1 h, homogenized in 2 ml of PBS, serially diluted, and plated on LB agar plates containing nalidixic acid. Additional biopsies were fixed by immersion in 10% buffered formalin, processed for paraffin embedding, cut and stained with hematoxylin and eosin. Histopathologic changes including hemorrhage, neutrophilic infiltration, edema, and necrosis and/or apoptosis were scored from 0 to 3 (0 for absence of lesions, and 1, 2, or 3 for mild, moderate, or severe lesion, respectively) for a combined total score ranging from 0 to 12.

### Mouse colitis model

Animals were obtained from The Jackson Laboratory (Bar Harbor), housed under specific-pathogen-free conditions and provided with water and food *ad libitum*. Groups of female, 9–12 week old C57BL/6 mice were orally treated with 20 mg Streptomycin. After 24 h, these mice were inoculated as described previously [61] with either 0.1 ml LB broth (mock treatment) or 1×10^9^ CFU of the *S.* Typhimurium SL1344 *fliC fljB phoN* mutant (SW793), the *fliC fljB phoN*::*tviA* mutant (SW764), the *fliC fljB phoN invA* mutant (SW794), or the *fliC fljB phoN*::*tviA invA* mutant (SW766) suspended in 0.1 ml LB broth. 12 h after infection, animals were euthanized and tissues were collected. The bacterial load was determined by spreading serial 10-fold dilutions of homogenates on LB agar plates containing the appropriate antibiotics. Flash-frozen cecal tissue was homogenized in a Mini-beadbeater (Biospec Products) and RNA was extracted by the TRI reagent method (Molecular Research Center). cDNA was generated using MuLV reverse transcriptase and reverse transcription reagents (Life Technologies). SYBR Green (Life Technologies)-based real-time PCR was performed as described previously [72] using the primers listed in table 3. Data was acquired by a ViiA 7 real-time PCR system (Life Technologies) and analyzed using the comparative Ct method (ΔΔ*Ct* method). Murine target gene transcription within each sample was normalized to the respective levels of *Gapdh* mRNA.

### Statistical analysis

Data obtained from tissue culture experiments, bacterial gene transcription experiments, and the bovine ligated ileal loop model was log-transformed prior to analysis with a paired Student's *t*-test. To determine statistical significance for relative mucosal mRNA transcription and tissue bacterial load between treatment groups, an unpaired Student's *t*-test was employed.

## Supporting Information

Figure S1 **TviA reduces T3SS-1-induced inflammatory responses independent of bacterial entry into host cells.** (A) Genetic organization of the *viaB* operon in *S.* Typhi Ty2. (B) The *S.* Typhi Ty2 wild-type strain, a Δ*viaB* mutant (SW347), a Δ*tviB-vexE* mutant (SW74), a Δ*viaB invA* mutant (STY4), and a Δ*tviB-vexE invA* mutant (SW611) cultured in DMEM were used to infect HeLa57 cells. NF-κB activation was determined after 5 h (N = 4). (C and D) HeLa 57A cells were infected with the *S.* Typhi Ty2 wild-type strain, a Δ*viaB* mutant (SW347), a Δ*viaB invA* mutant (STY4), or a Δ*viaB invA* mutant harboring pRI203 (N = 3) precultured in TYE broth. (C) Cells were infected at a multiplicity of infection of 5 for 1 h and extracellular bacteria killed by treatment with Gentamicin for 90 min. Recovered bacterial numbers were standardized to the number of the bacteria in the inoculum. (D) To determine NF-κB activation, luciferase activity measured 5 h after infection (N = 4). Bars represent geometric means ± standard error. **, *P*<0.01; ns, not statistically significant.
(TIF)

Figure S2 **TviA reduces T3SS-1-induced NF-κB activation independent of flagellin expression.** (A) Schematic representation of the TviA regulatory network in *S.* Typhi and effect on host signaling pathways. (B and C) HeLa 57A cells (B) or HEK-293 cells transiently transfected with a NF-κB-dependent reporter plasmid (pNFkB-luc) (C) were infected with a *S.* Typhi Δ*fliC* mutant (SW359), a Δ*fliC* Δ*viaB* mutant (SW483), derivatives carrying the cloning plasmids pWSK29 (pWSK) or the plasmid pTVIA1, and a Δ*fliC* Δ*viaB invA* mutant (SW398). Luciferase activity was quantified 5 h after infection to determine NF-κB activation levels (N = 3). Bars represent geometric means ± standard error. *, *P*<0.05; **, *P*<0.01; ***, *P*<0.001.
(TIF)

Figure S3 **Effect of TviA on bacterial gene expression** ***in vitro*** **.** The *S.* Typhi wild type Ty2 (WT), a Δ*viaB* mutant (SW347), a Δ*tviB-vexE* mutant (SW74), the *S.* Typhimurium wild-type SL1344, a SL1344 *phoN* mutant (SW759), a SL1344 *phoN*::*tviA* mutant (SW760), the *S.* Typhimurium 14028 Nal^R^ wild type (IR715), a 14028 Nal^R^ *phoN* mutant (AJB715), and a 14028 Nal^R^ *phoN*::*tviA* (SW474) were cultured in TYE broth for 3 h. RNA was extracted and qRT-PCR performed to determine the relative abundance of *flhD* (A), *hilA* (B), *invF* (C), *prgH* (D), *sipA* (E), and *sopE* (F) mRNA. Data presented is fold change over the abundance of mRNA recovered from the respective wild-type strain after standardization to the housekeeping gene *gmk*. The dotted line indicates no change in gene expression. Bars represent geometric means from 3 (*S.* Typhimurium) or 4 (*S.* Typhi) independent experiments ± standard error. *, *P*<0.05; **, *P*<0.01; ***, *P*<0.001; ns, not statistically significant.
(TIF)

Figure S4 **Contribution of SopE, SipA, SopB, and SopE2 to NF-κB activation in human epithelial cells.** HeLa 57A cells were treated with media only (mock treatment) or infected with the indicated *S.* Typhimurium SL1344 derivatives. Certain *Salmonella* strains lacked defined T3SS-1 effector proteins to analyze the responses induced by SopB (light grey bar), SipA (white bar), and SopE (dark grey bar). NF-κB activation was assessed 5 h after infection based on a NF-κB-driven luciferase reporter system (N = 4). Bars represent geometric means ± standard error. **, *P*<0.01; ns, not statistically significant.
(TIF)

Figure S5 **Inhibition of RIP2 signaling does not affect invasiveness of** ***S.*** **Typhi strains.** HeLa 57A cells pretreated with DMSO or RIP2 inhibitor (SB203580; dissolved in DMSO) were infected with the indicated *S.* Typhi strains at a MOI of 10 and invasion determined by a Gentamicin protection assay. Bars represent geometric means ± standard error. ns, not statistically significant.
(TIF)

Figure S6 **Expression of TviA in** ***S.*** **Typhimurium 14028 Nal^R^ reduces T3SS-1-driven NF-κB activation in epithelial cells.** HeLa 57A cells were infected with the *S.* Typhimurium 14028 Nal^R^ derivatives or treated with bacterial growth media (mock treatment). Luciferase activity determined 5 h after infection (N = 4). Bars represent geometric means ± standard error. **, *P*<0.01;
(TIF)

Figure S7 **Bacterial colonization in the mouse colitis model.** (A and B) Streptomycin-pretreated mice were infected with the indicated *S.* Typhimurium SL1344 derivatives as described in Figure 6. The bacterial load in the colon content (A) and the Peyer's patches (B) was determined 12 h after infection. Bars represent geometric means ± standard error. ns, not statistically significant.
(TIF)
