# Supplementary figures and images for: Salmonella enterica Serovar Typhi Conceals the Invasion-Associated Type Three Secretion System from the Innate Immune System by Gene Regulation
Source: PLoS Pathog. 2014 Jul 3;10(7):e1004207. doi: 10.1371/journal.ppat.1004207 (PMC4081808; doi:10.1371/journal.ppat.1004207)

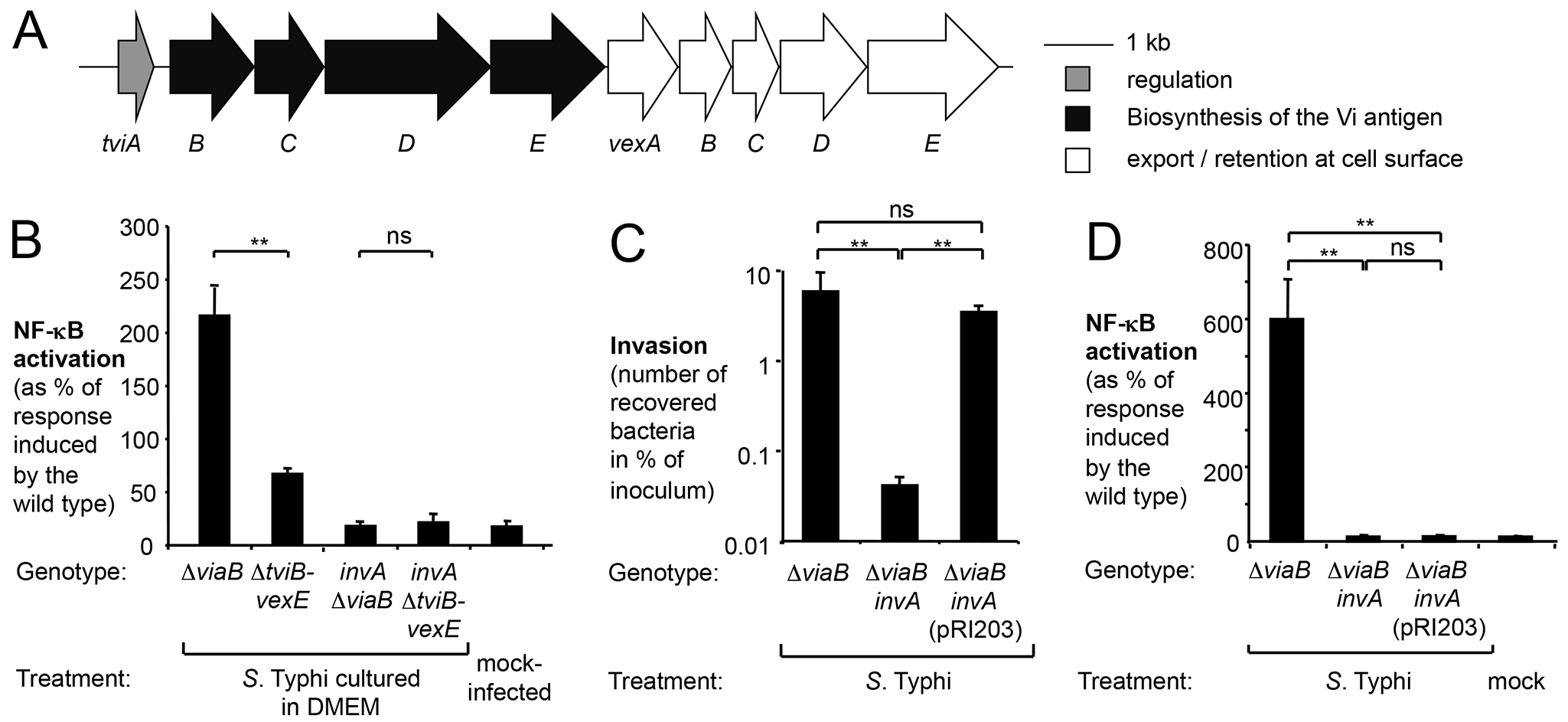

Supplement: Figure S1 — TviA reduces T3SS-1-induced inflammatory responses independent of bacterial entry into host cells. (A) Genetic organization of the viaB operon in S. Typhi Ty2. (B) The S. Typhi Ty2 wild-type strain, a ΔviaB mutant (SW347), a ΔtviB-vexE mutant (SW74), a ΔviaB invA mutant (STY4), and a ΔtviB-vexE invA mutant (SW611) cultured in DMEM were used to infect HeLa57 cells. NF-κB activation was determined after 5 h (N = 4). (C and D) HeLa 57A cells were infected with the S. Typhi Ty2 wild-type strain, a ΔviaB mutant (SW347), a ΔviaB invA mutant (STY4), or a ΔviaB invA mutant harboring pRI203 (N = 3) precultured in TYE broth. (C) Cells were infected at a multiplicity of infection of 5 for 1 h and extracellular bacteria killed by treatment with Gentamicin for 90 min. Recovered bacterial numbers were standardized to the number of the bacteria in the inoculum. (D) To determine NF-κB activation, luciferase activity measured 5 h after infection (N = 4). Bars represent geometric means ± standard error. **, P<0.01; ns, not statistically significant. (TIF) [file ppat.1004207.s001.tif]

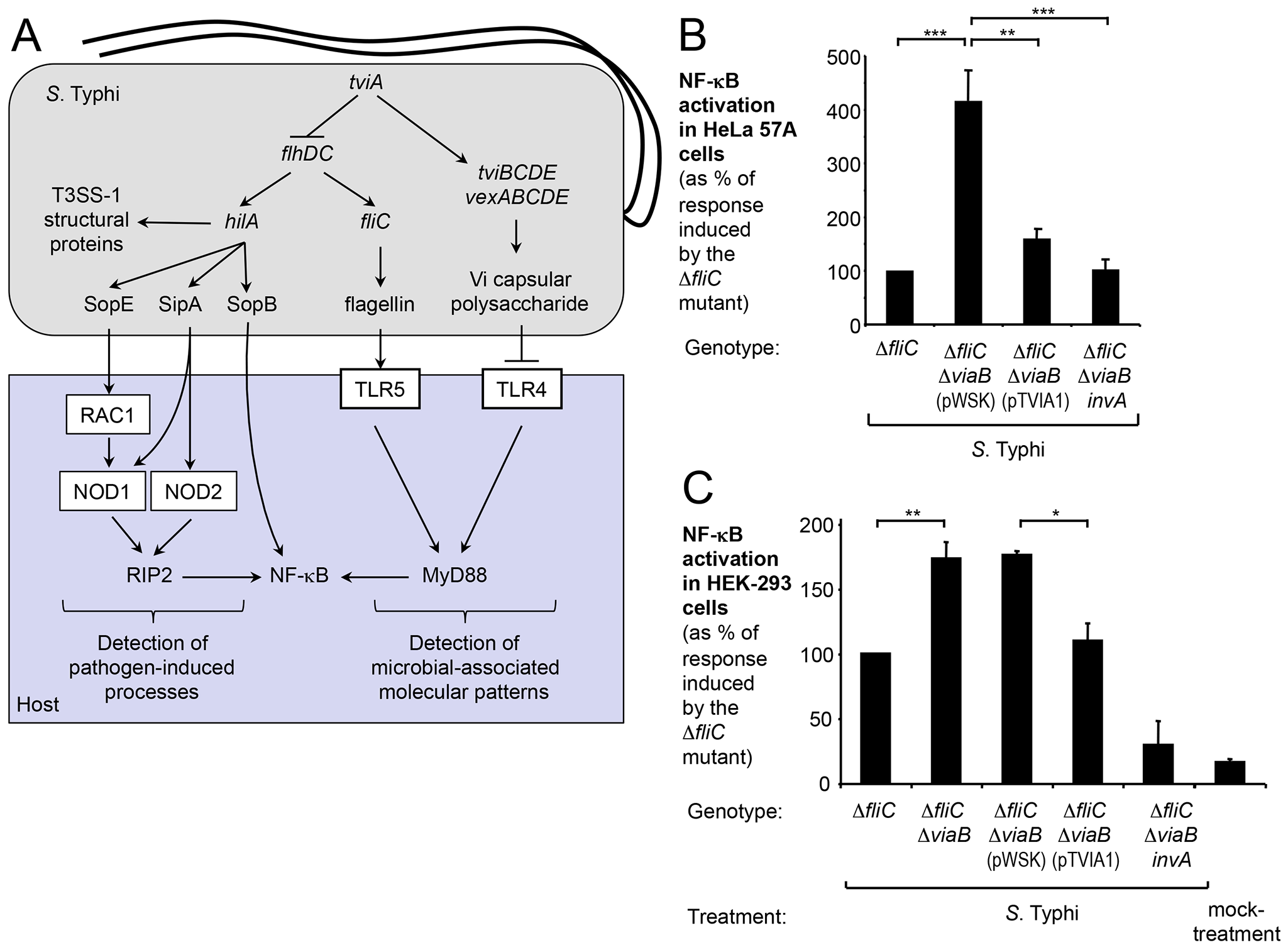

Supplement: Figure S2 — TviA reduces T3SS-1-induced NF-κB activation independent of flagellin expression. (A) Schematic representation of the TviA regulatory network in S. Typhi and effect on host signaling pathways. (B and C) HeLa 57A cells (B) or HEK-293 cells transiently transfected with a NF-κB-dependent reporter plasmid (pNFkB-luc) (C) were infected with a S. Typhi ΔfliC mutant (SW359), a ΔfliC ΔviaB mutant (SW483), derivatives carrying the cloning plasmids pWSK29 (pWSK) or the plasmid pTVIA1, and a ΔfliC ΔviaB invA mutant (SW398). Luciferase activity was quantified 5 h after infection to determine NF-κB activation levels (N = 3). Bars represent geometric means ± standard error. *, P<0.05; **, P<0.01; ***, P<0.001. (TIF) [file ppat.1004207.s002.tif]

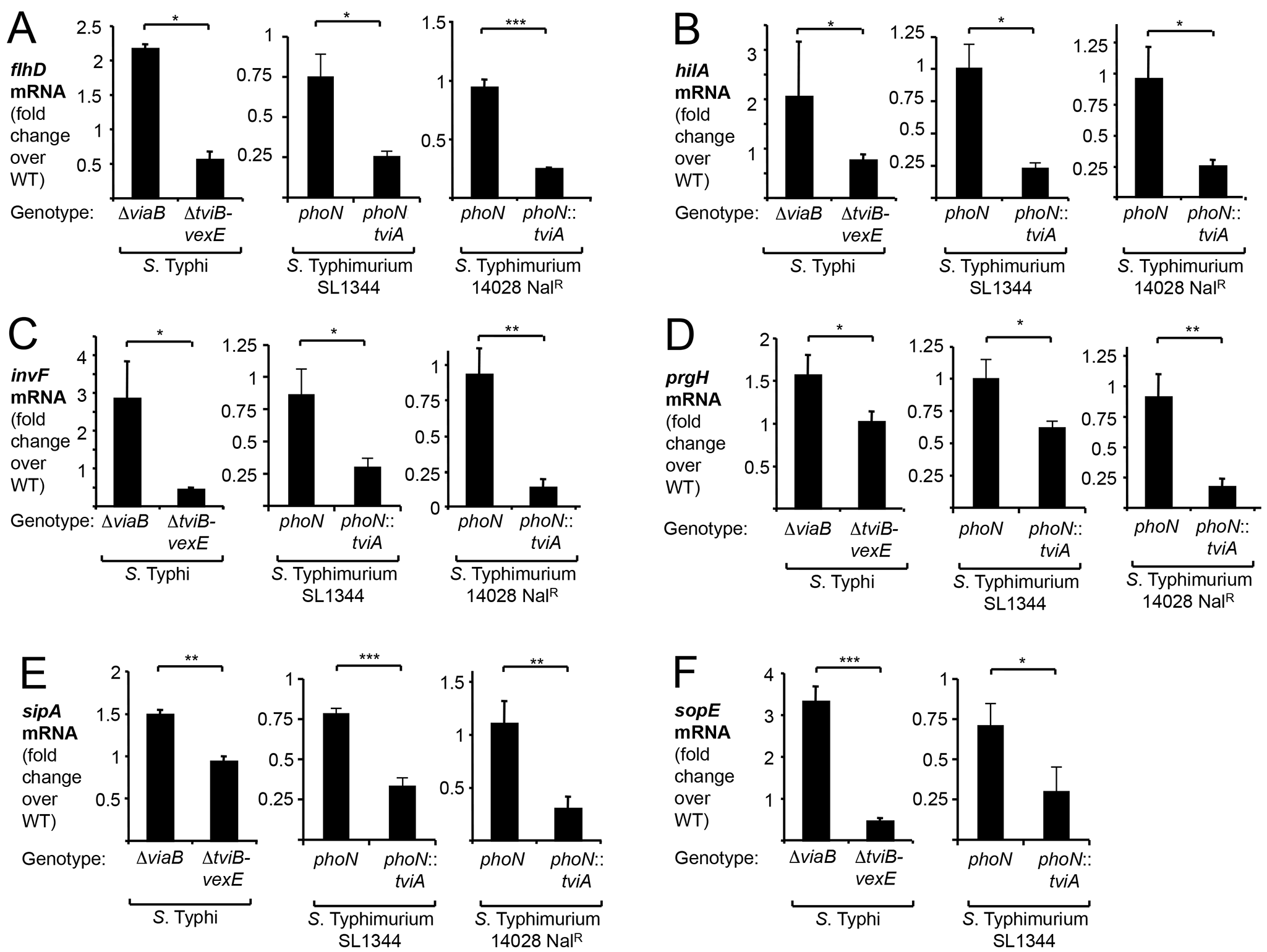

Supplement: Figure S3 — Effect of TviA on bacterial gene expression in vitro . The S. Typhi wild type Ty2 (WT), a ΔviaB mutant (SW347), a ΔtviB-vexE mutant (SW74), the S. Typhimurium wild-type SL1344, a SL1344 phoN mutant (SW759), a SL1344 phoN::tviA mutant (SW760), the S. Typhimurium 14028 NalR wild type (IR715), a 14028 NalR phoN mutant (AJB715), and a 14028 NalR phoN::tviA (SW474) were cultured in TYE broth for 3 h. RNA was extracted and qRT-PCR performed to determine the relative abundance of flhD (A), hilA (B), invF (C), prgH (D), sipA (E), and sopE (F) mRNA. Data presented is fold change over the abundance of mRNA recovered from the respective wild-type strain after standardization to the housekeeping gene gmk. The dotted line indicates no change in gene expression. Bars represent geometric means from 3 (S. Typhimurium) or 4 (S. Typhi) independent experiments ± standard error. *, P<0.05; **, P<0.01; ***, P<0.001; ns, not statistically significant. (TIF) [file ppat.1004207.s003.tif]

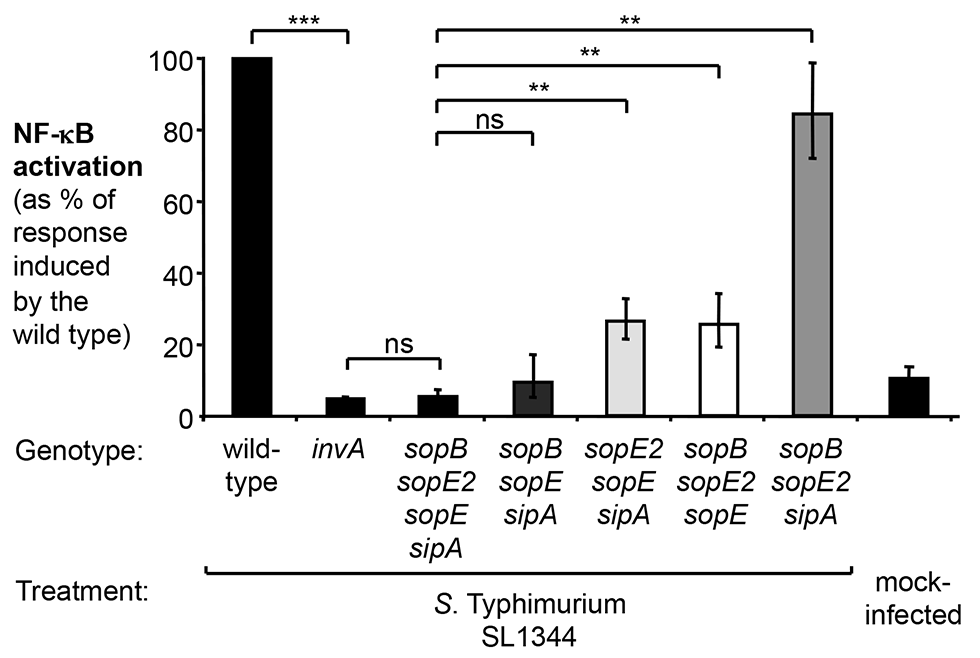

Supplement: Figure S4 — Contribution of SopE, SipA, SopB, and SopE2 to NF-κB activation in human epithelial cells. HeLa 57A cells were treated with media only (mock treatment) or infected with the indicated S. Typhimurium SL1344 derivatives. Certain Salmonella strains lacked defined T3SS-1 effector proteins to analyze the responses induced by SopB (light grey bar), SipA (white bar), and SopE (dark grey bar). NF-κB activation was assessed 5 h after infection based on a NF-κB-driven luciferase reporter system (N = 4). Bars represent geometric means ± standard error. **, P<0.01; ns, not statistically significant. (TIF) [file ppat.1004207.s004.tif]

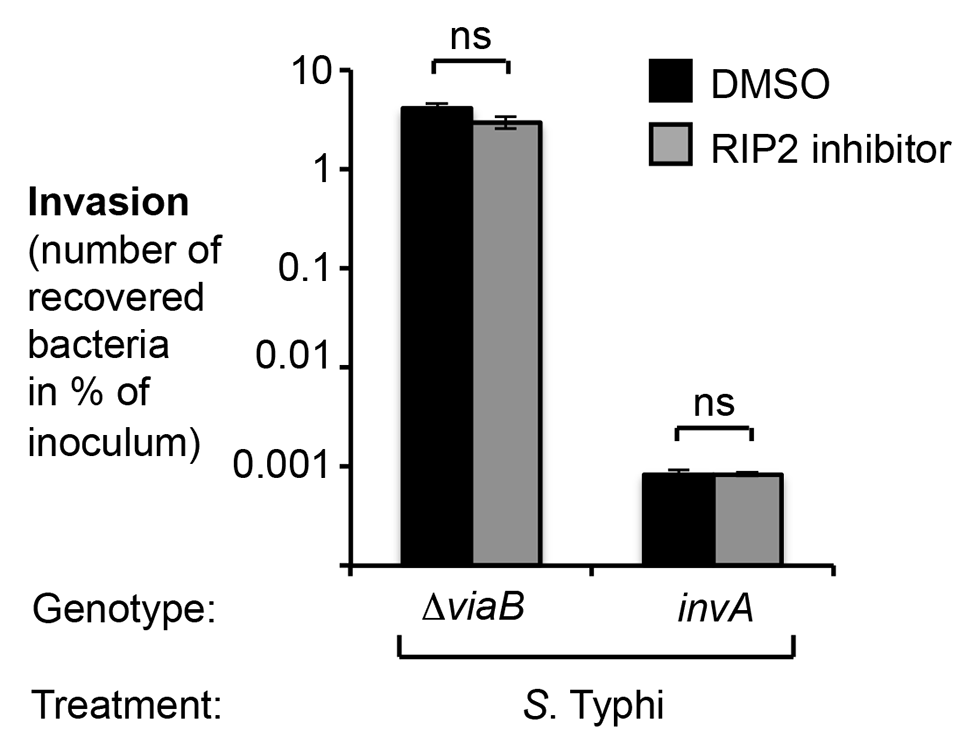

Supplement: Figure S5 — Inhibition of RIP2 signaling does not affect invasiveness of S. Typhi strains. HeLa 57A cells pretreated with DMSO or RIP2 inhibitor (SB203580; dissolved in DMSO) were infected with the indicated S. Typhi strains at a MOI of 10 and invasion determined by a Gentamicin protection assay. Bars represent geometric means ± standard error. ns, not statistically significant. (TIF) [file ppat.1004207.s005.tif]

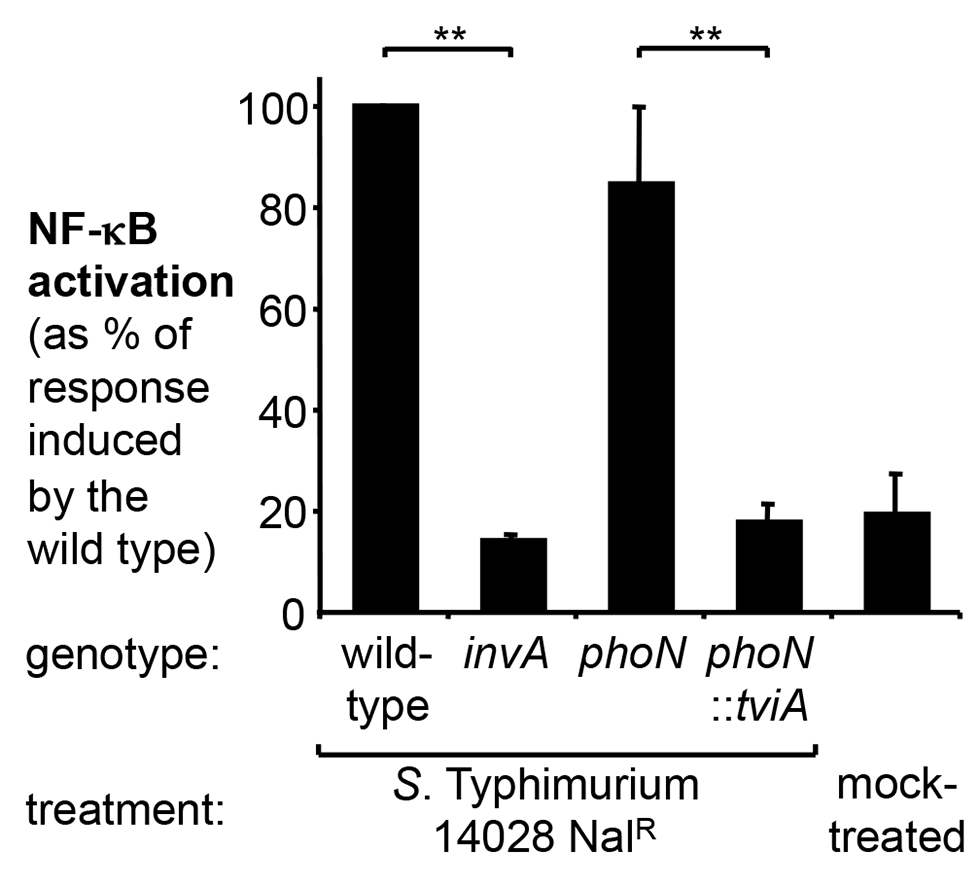

Supplement: Figure S6 — Expression of TviA in S. Typhimurium 14028 NalR reduces T3SS-1-driven NF-κB activation in epithelial cells. HeLa 57A cells were infected with the S. Typhimurium 14028 NalR derivatives or treated with bacterial growth media (mock treatment). Luciferase activity determined 5 h after infection (N = 4). Bars represent geometric means ± standard error. **, P<0.01; (TIF) [file ppat.1004207.s006.tif]

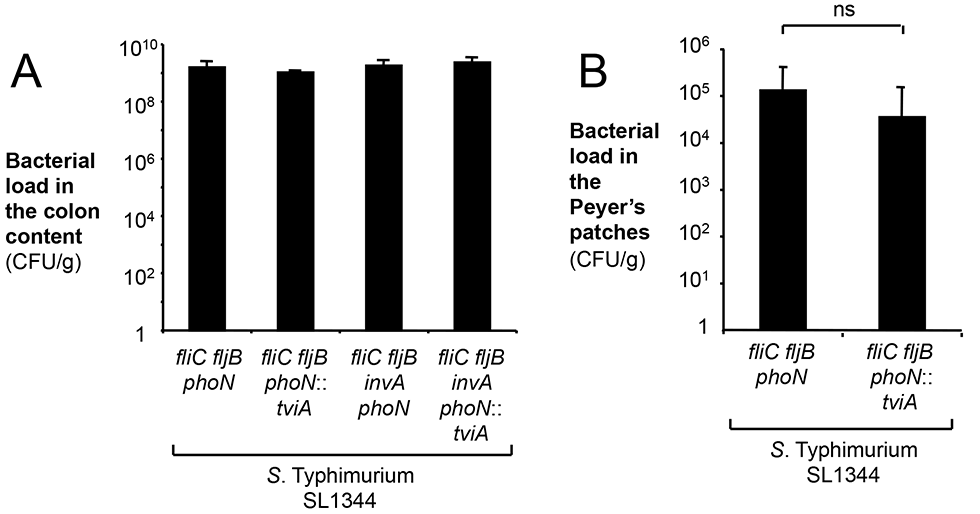

Supplement: Figure S7 — Bacterial colonization in the mouse colitis model. (A and B) Streptomycin-pretreated mice were infected with the indicated S. Typhimurium SL1344 derivatives as described in Figure 6. The bacterial load in the colon content (A) and the Peyer's patches (B) was determined 12 h after infection. Bars represent geometric means ± standard error. ns, not statistically significant. (TIF) [file ppat.1004207.s007.tif]
